# Estimating the mean in the space of ranked phylogenetic trees

**DOI:** 10.1093/bioinformatics/btae514

**Published:** 2024-08-23

**Authors:** Lars Berling, Lena Collienne, Alex Gavryushkin

**Affiliations:** Biological Data Science Lab, School of Mathematics and Statistics, University of Canterbury, Christchurch 8041, New Zealand; Biological Data Science Lab, School of Mathematics and Statistics, University of Canterbury, Christchurch 8041, New Zealand; Biological Data Science Lab, School of Mathematics and Statistics, University of Canterbury, Christchurch 8041, New Zealand

## Abstract

**Motivation:**

Reconstructing evolutionary histories of biological entities, such as genes, cells, organisms, populations, and species, from phenotypic and molecular sequencing data is central to many biological, palaeontological, and biomedical disciplines. Typically, due to uncertainties and incompleteness in data, the true evolutionary history (phylogeny) is challenging to estimate. Statistical modelling approaches address this problem by introducing and studying probability distributions over all possible evolutionary histories, but can also introduce uncertainties due to misspecification. In practice, computational methods are deployed to learn those distributions typically by sampling them. This approach, however, is fundamentally challenging as it requires designing and implementing various statistical methods over a space of phylogenetic trees (or treespace). Although the problem of developing statistics over a treespace has received substantial attention in the literature and numerous breakthroughs have been made, it remains largely unsolved. The challenge of solving this problem is 2-fold: a treespace has nontrivial often counter-intuitive geometry implying that much of classical Euclidean statistics does not immediately apply; many parametrizations of treespace with promising statistical properties are computationally hard, so they cannot be used in data analyses. As a result, there is no single conventional method for estimating even the most fundamental statistics over any treespace, such as mean and variance, and various heuristics are used in practice. Despite the existence of numerous tree summary methods to approximate means of probability distributions over a treespace based on its geometry, and the theoretical promise of this idea, none of the attempts resulted in a practical method for summarizing tree samples.

**Results:**

In this paper, we present a tree summary method along with useful properties of our chosen treespace while focusing on its impact on phylogenetic analyses of real datasets. We perform an extensive benchmark study and demonstrate that our method outperforms currently most popular methods with respect to a number of important ‘quality’ statistics. Further, we apply our method to three empirical datasets ranging from cancer evolution to linguistics and find novel insights into corresponding evolutionary problems in all of them. We hence conclude that this treespace is a promising candidate to serve as a foundation for developing statistics over phylogenetic trees analytically, as well as new computational tools for evolutionary data analyses.

**Availability and implementation:**

An implementation is available at https://github.com/bioDS/Centroid-Code.

## 1 Introduction

Reconstructing evolutionary (or phylogenetic) trees from sequence data is an important task across a variety of areas including evolutionary biology, epidemiology, developmental biology, palaeontology, and linguistics. For this task, there exist four main tree inference paradigms that are based on different analytical approaches (Felsenstein 2004): sequence (dis)similarity, parsimony, maximum likelihood, and Bayesian. Distance (or sequence dissimilarity) based tree reconstruction approaches, including the very popular Neighbour Joining algorithm (Saitou and Nei 1987), infer trees from a distance matrix between sequences (typically multiple sequence alignments obtained from DNA, RNA, or proteins). Parsimony based approaches are aimed at finding the tree that minimizes the number of changes along its branches given sequence data (Fitch 1971, Swofford 1999) at the tips. Maximum likelihood based tree reconstruction approaches are sampling the likelihood surface over all possible trees with the aim to find the tree (and branch lengths) that maximizes the likelihood given sequence data and an evolutionary model (Felsenstein 1981, Stamatakis 2014, Nguyen *et al.* 2015). Bayesian inference approaches are aimed at sampling (usually using Markov Chain Monte Carlo algorithms) the joint posterior probability distribution of parameters of interest, typically including the tree parameter (Huelsenbeck and Ronquist 2001, Drummond and Rambaut 2007, Höhna *et al.* 2016, Bouckaert *et al.* 2019).

In probability theory, uncertainties over parameter estimates arise from the fact that data are finite. In practice when phylogenetic trees are inferred from sequence data, additional factors come into play, such as alignment errors, substitution model (mis)specification, (nontree) model parameter estimation errors, sampling limitations, sequencing quality, site-wise ambiguities in phylogenetic signal, etc. Because the approach we develop in this paper is applicable in both theoretical and applied phylogenetics, we abuse the notation here and refer to all these sources of error as ‘uncertainty’. This convention is consistent with common practice when, due to the fact that it is hard or impossible to correctly identify the source of ‘uncertainty’, all these factors are consolidated into a single measure that is reported in analyses, although exceptions exist (Chen *et al.* 2022). Two typical computational examples include bootstrapping in the maximum likelihood or posterior support in the Bayesian framework (Efron *et al.* 1996). Other examples of when reporting a single tree is inadequate include equally parsimonious trees (Maddison 1991), trees with indistinguishably similar likelihoods (Steel 1994), and phylogenetic terraces (Sanderson *et al.* 2011, 2015).

To overcome these issues in a statistically sound way, Holmes (1999) initiated a research programme aimed at developing a mathematical framework for statistical analyses over the space of phylogenetic trees. The programme has since received significant attention in the literature with a number of promising treespaces introduced (Kim 2000, Billera *et al.* 2001, Moulton and Steel 2004, Gill *et al.* 2008, Gavryushkin and Drummond 2016, Kendall and Colijn 2016, Lin *et al.* 2017, Gavryushkin *et al.* 2018, Feragen and Nye 2020, Collienne *et al.* 2021, Garba *et al.* 2021) and a range of statistical methods developed (Holmes 2003, Nye 2011, Bacák 2014, Benner *et al.* 2014, Miller *et al.* 2015, Nye 2020, Lin *et al.* 2018, Willis and Bell 2018, Willis 2019, Yoshida *et al.* 2019, Page *et al.* 2020). However, the ultimate milestone of the original programme of introducing fundamental statistics, such as the mean, variance, confidence intervals, for probability distributions over any treespace in a way that would enable a wide range of practical applications remains illusive (McMorris and Steel 1994, Heled and Bouckaert 2013, Hotz *et al.* 2013, Lin *et al.* 2017).

One idea that cuts through the many successes so far was that of using geometric properties of treespace to introduce statistical notions. Indeed, this idea was developed by Billera *et al.* (2001) for treespaces, where means of probability distributions over the treespace have been defined as geometric means, i.e. as trees that minimize the sum of squared distances. Matsen (2006) extended this idea to shapes. The definition of variance in this case follows naturally, and this approach has been developed in a number of treespaces (Barthélemy and McMorris 1986, Heled and Bouckaert 2013, Bacák 2014, Benner *et al.* 2014, Miller *et al.* 2015). Geometric ideas (Owen and Provan 2011, Bacák 2014, Miller *et al.* 2015) enabled an algorithm based on the idea of (Sturm 2002) to estimate the mean of a set of trees. Nye (2011) developed a construction of the first principle component, a milestone for statistical inference in treespaces, with a version developed for tropical geometric interpretation of treespace in Yoshida *et al.* (2019) and Page *et al.* (2020). In addition, Barden *et al.* (2013) were able to prove a Central Limit Theorem in the BHV (Billera *et al.* 2001) treespace with four taxa and Nye (2020) constructed random walks over this treespace and presented approaches to define uncertainty for collections of phylogenetic trees (Willis and Bell 2018, Willis 2019).

These results exposed the main complications that need to be overcome to enable statistically sound and practically useful tree inference methods. These include the stickiness of the mean (Hotz *et al.* 2013) (irresponsiveness of the mean tree to changes in the tree sample), prevalence of star-like mean trees even for phylogenetically informative data (Hotz *et al.* 2013, Gavryushkin and Drummond 2016), stickiness of the first principal component (Nye 2011, Feragen and Nye 2020), the high dimensionality problem for small samples of trees (Lin *et al.* 2017), and others (Lin *et al.* 2018). Some of these complications are actively being addressed with, e.g. the Wald space (Garba *et al.* 2021, Lueg *et al.* 2021) being proposed to tackle the mean stickiness issue. These, however, still do not address the problem in full and as of today it is still common practice to use heuristic methods that focus on finding consensus among a given set of trees (Bryant 2003, Heled and Bouckaert 2013, Brown and Owen 2020). Partly, this is due to the fact that in treespaces where geometric summary trees were hoped to be phylogenetically informative, such as Robinson–Foulds (Robinson and Foulds 1979, Barthélemy and McMorris 1986), NNI (Robinson 1971), and SPR (Whidden and Matsen 2015), they are NP-hard to compute (McMorris and Steel 1994, DasGupta *et al.* 1998, Bordewich and Semple 2005) and have hardly received adoption into practice, despite the availability of practical algorithms (Bansal *et al.* 2010, Whidden *et al.* 2013) for small trees and/or distances. In addition, recent results suggest counter-intuitive behaviour of these popularly used metrics (Smith 2020, 2022).

Motivated by the research programme initiated by Holmes (1999) and Gavryushkin and Drummond (2016) investigated whether the treespaces developed thus far were readily adaptable to phylogenetic trees scaled to time, which is an important class called time trees with many popular inference methods, such as UPGMA (Sokal 1958), BEAST (Drummond and Rambaut 2007), and BEAST2 (Bouckaert *et al.* 2019). Surprisingly, they discovered that the space of time trees has unique characteristics and is significantly different, geometrically and algorithmically, from the space of classical phylogenetic trees. Along this line of research, it has recently been established that a discrete component of time trees, the RNNI (Ranked Nearest Neighbour Interchange) space (Gavryushkin *et al.* 2018), which is based on tree rearrangement operations from Markovtsova *et al.* (2000), is computationally tractable (Collienne and Gavryushkin 2021), unlike its classic version the NNI space (DasGupta *et al.* 1998). Furthermore, it was shown that the RNNI space possesses several characteristics indicating its suitability for statistical approaches (Collienne *et al.* 2021). For example, this space satisfies the cluster property (Collienne *et al.* 2021), which requires a cluster shared by two trees to be also shared by all trees on shortest paths between the two trees. This property is important because it implies that information shared by trees in a sample is preserved in the summary tree. Our results here will justify this further.

In this paper, we continue this line of research and contribute to the programme by demonstrating that for time trees it is possible to introduce fundamental statistics in a way that resolves the main problems (outlined above) to which all popular approaches are prone. We demonstrate that for time trees the notion of a mean can be introduced and computationally approximated in a way that is statistically sound, practically computable for large datasets, and an improvement over known approaches commonly used in practice. We carry out a simulation study to validate our method and compare it to other tree summary approaches such as the Maximum Clade Credibility (MCC) method. As part of this study, we have developed an approach to assess suitability of a treespace for statistical analyses, e.g. its ‘smoothness’ with respect to probability distributions over trees. Finally, we apply our method to three real datasets from previous studies of Dravidian languages (linguistics), Weevils (phylogeography), and cancer development (within-organism somatic evolution). Interestingly, in all three cases the summary tree obtained using our methods, although different from the ones published, is consistent with the corresponding evolutionary processes and discussions in the original studies. Combined, these considerations imply that the approach introduced in this paper is a promising candidate to become a mathematical foundation for statistical analyses in the space of phylogenetic time trees.

## 2 Materials and methods

### 2.1 RNNI space

**Figure 1.**
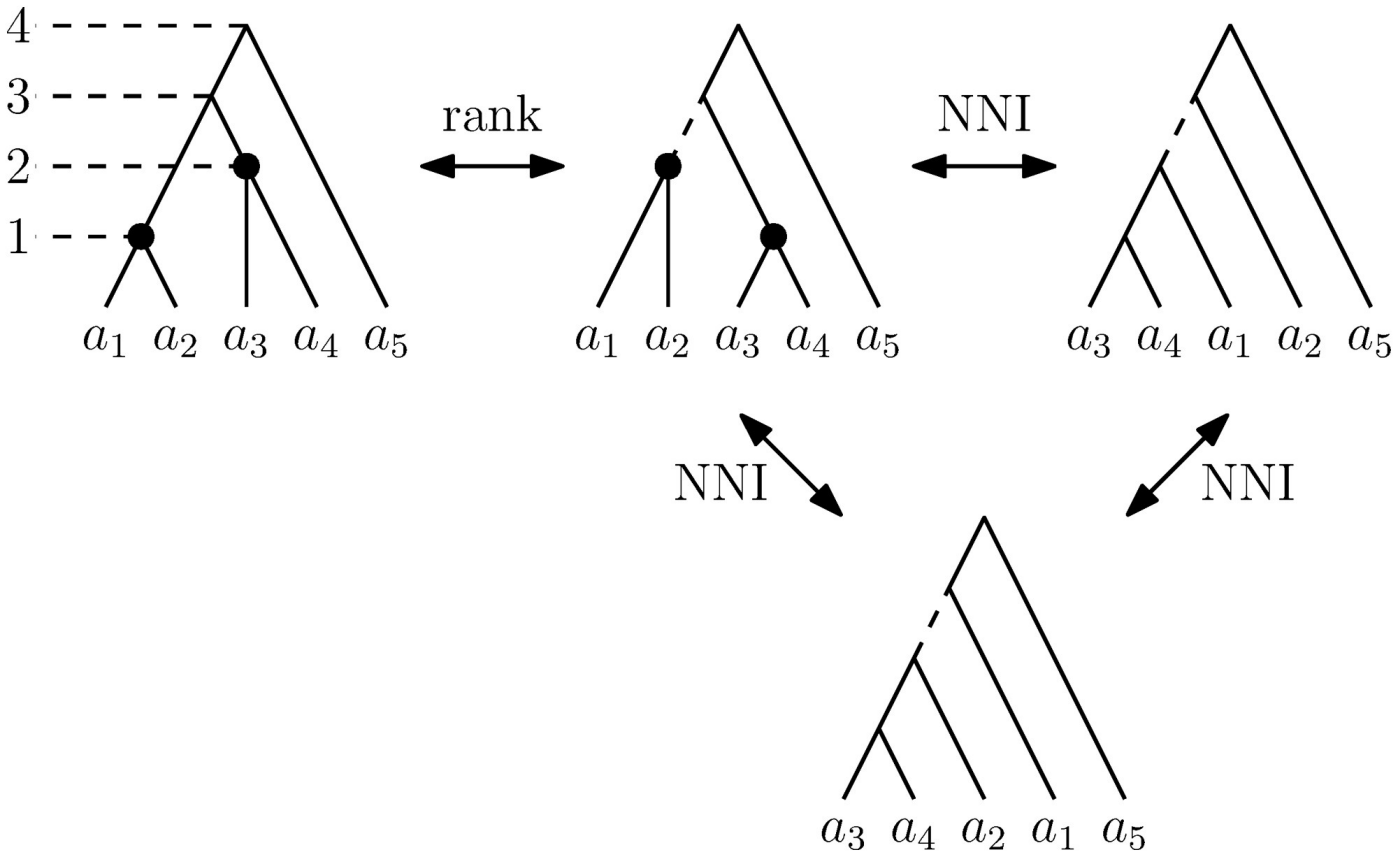
An illustration of trees with five taxa in the RNNI treespace.

The RNNI space (Gavryushkin *et al.* 2018) is a treespace of ranked phylogenetic trees, which are rooted binary trees where internal nodes are ordered according to times of the corresponding evolutionary events, assuming no co-occurrence. In other words, every internal node in a ranked tree with *n* leaves is assigned a unique integer (rank) between 1 and *n* − 1 such that no node has rank higher than its parent, and all leaves are assumed to have rank 0. The RNNI space is then defined as a graph where vertices are ranked trees and edges are representing either a rank or an NNI move that transforms one tree into another. A rank move swaps the order of two unconnected nodes with consecutive ranks (i.e. ranks *i* and *i *+* *1) in a tree. An NNI move can be performed on an edge in a tree that connects two nodes with consecutive ranks by moving either of the two sister clades adjacent to the bottom node to the opposite side of the top node. The RNNI distance between trees is then defined as the length of a shortest path in this graph, that is the minimal number of rank and NNI moves to transform one tree into another. See Fig. 1 for an illustration of the RNNI space. This distance is efficiently computable (Collienne and Gavryushkin 2021), which enables computational tools necessary for our approaches to be applicable in practice. The RNNI treespace is the main tool used in this paper.

### 2.2 Mean tree

In the following, we present an algorithm to approximate the mean (defined as a centroid) tree for a set of ranked trees in the RNNI space. The work of Sturm (2002) gives an iterative algorithm to approximate the mean and variance of a probability distribution over a nonpositively curved metric space, which is directly applicable to treespaces such as the BHV (Bacák 2014, Miller *et al.* 2015, Brown and Owen 2020) and *τ*-space (Gavryushkin and Drummond 2016). As a consequence, basic probability theory results such as the Law of Large Numbers, which states that the sample mean converges to the true mean, hold in these spaces (Sturm 2002). Importantly, the results of Sturm (2002) cannot be directly applied to the RNNI space because this space has positive curvature (Gavryushkin *et al.* 2018). To adapt these approaches to positively curved geodesic metric spaces in this paper, we introduce the Centroid algorithm (Algorithm 1) that uses local search to minimize the sum of squared (SoS) distances, also called a Fréchet variance, between a summary tree and a given tree sample and stops when it finds a locally optimal tree *T_a_*, approximating a centroid tree $T^{*}$.

More formally, the input to the Centroid algorithm (Algorithm 1) is a set of trees T and a starting tree *T_start_*, which can generally be chosen arbitrarily but is computed by an adaptation of a known tree summary method in our case (see Supplementary Section S2.2.1 and Supplementary Material for a discussion on this topic). In the first step, $T_{a}:=T_{\mathrm{start}}$ is used as approximation of the mean tree and the SoS value for *T_start_* is computed. The algorithm proceeds iteratively by computing the SoS values for all neighbours of the current tree *T_a_* (i.e. trees with RNNI distance one to *T_a_*)—this step can be executed in parallel for each neighbour. Then *T_a_* gets updated to be the neighbour with lowest SoS value (if there are multiple such neighbours, one is chosen randomly) and we proceed to the next iteration. If there is no neighbour with lower SoS value than *T_a_*, a local optimum is reached and *T_a_* is returned as approximation of the mean tree for the set T. We provide an implementation of this algorithm in the following github repository: https://github.com/bioDS/Centroid-Code.

Algorithm 1 Centroid

** procedure** Centroid(tree *T_start_*, tree set T)

  $T_{a}\leftarrow T_{\mathrm{start}}$

  **while**$\emptyset \neq N\leftarrow \{N\;\text{neighbour of}\;T_{a}:$${\text{SoS}}_{T}(N)<{\text{SoS}}_{T}(T_{a})\}$   **do**:

   $T_{a}\leftarrow \underset{N\in N}{\text{arg min}}{\text{SoS}}_{T}(N)$

**  end whilereturn** *T_a_*

** end procedure**

### 2.3 Avoiding nonglobal optima

The Centroid algorithm (Algorithm 1) requires a starting tree as input. Choosing a good initial tree is important for both the accuracy of the algorithm (the quality of the approximation) and the running time. For computing a starting tree for the Centroid algorithm we use the algorithmic idea presented by Sturm (2002), which is used in Miller *et al.* (2015) to approximate mean trees in BHV-space and in Gavryushkin and Drummond (2016) to approximate the mean in *τ*-space. It was shown by Sturm (2002) that this algorithm converges to the true mean under the Law of Large Numbers, and as we’ll see below it provides a good initial starting tree for our approximation algorithm (see Supplementary Section S5.1 for a comparison to other choices).

Our version of the Sturm algorithm adapted to the RNNI treespace works as follows. In the first iteration, we select a tree *T* uniformly at random from the set of trees T and remove it from this set. In every following iteration k=2,…,m we choose a tree *R* uniformly at random and remove it from T. We then compute a shortest path *p* from *R* to *T*, using the FindPath algorithm (Collienne and Gavryushkin 2021). The tree *T* for the next iteration is then defined as the tree at position $\left\lfloor \frac{1}{k}\right\rfloor$ of *p*. Note that this implies that if $\left\lfloor \frac{1}{k}\right\rfloor =0$, the tree *T* does not change. The time complexity of this procedure is in $O(|T|*n^{2})$ where $O(n^{2})$ is the complexity of calculating the necessary tree on the shortest path between the two trees in RNNI (Collienne and Gavryushkin 2021).

### 2.4 Example

**Figure 2.**
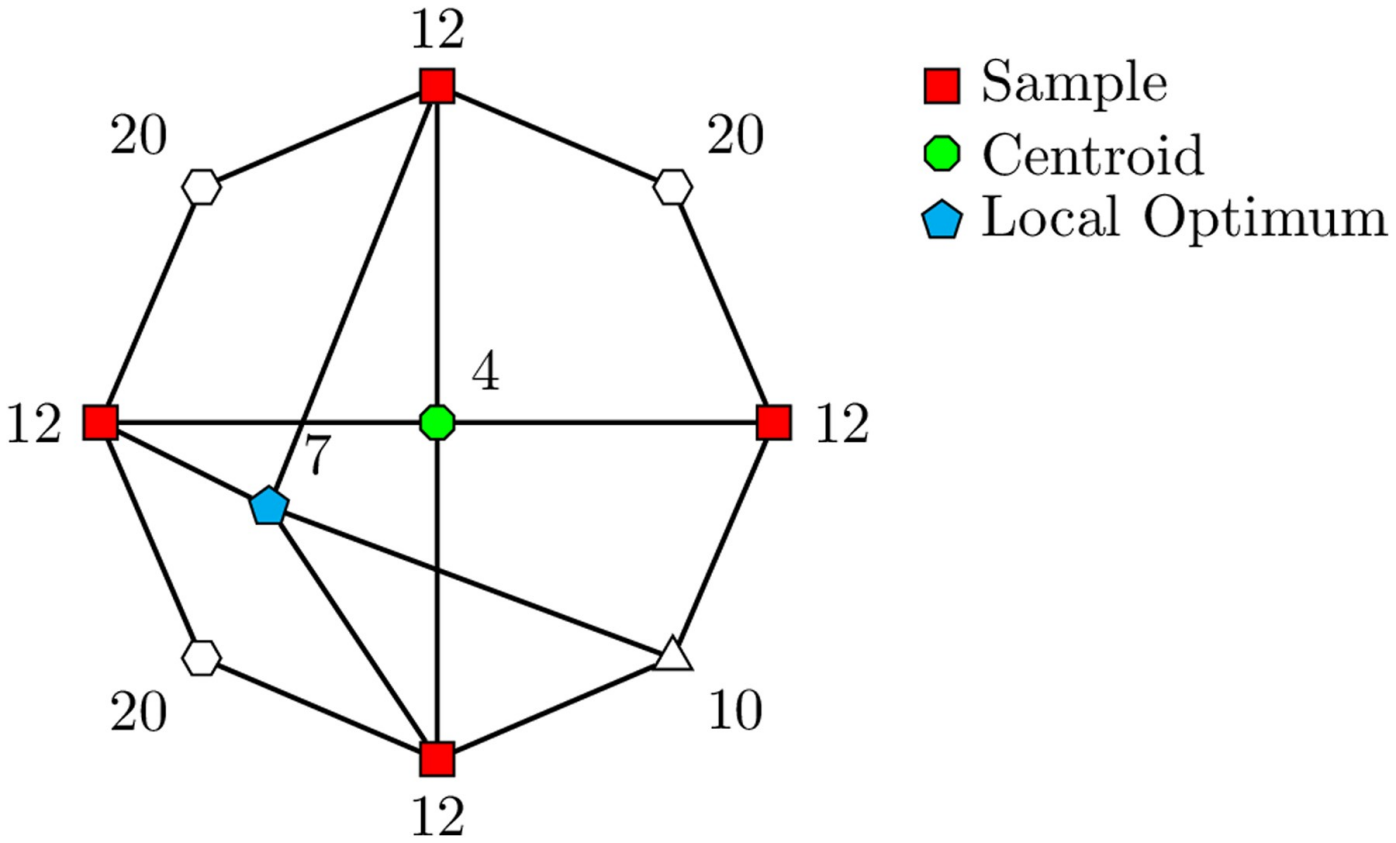
Simplified illustration of a treespace (entire graph) featuring a subset of trees (red squares). Each vertex label represents its SoS value within this sample of trees. The graph highlights one globally optimal tree (green octagon) and a locally optimal tree (cyan pentagon), both discoverable via the Centroid algorithm, dependent on the starting tree selection.

In this context, we present a representative example in Fig. 2 of the unique challenges that our algorithm faces within the RNNI treespace. The graph does not depict a real treespace; rather, we assume it to be representative of such. Each node in the graph represents a tree, and each edge denotes either a rank or an NNI move. We identify a subset of nodes (red squares) representing a set of trees used as input for our Centroid algorithm. In addition, each vertex is labelled with its SoS value. We highlight the globally optimal tree, or centroid (green octagon), and a locally optimal tree (cyan pentagon). Notably, due to symmetry, three of the remaining vertices are white hexagons, and one is a white triangle. This example illustrates that the algorithm’s outcome may vary depending on its starting tree, potentially resulting in a locally optimal tree rather than the global optimum.

### 2.5 Annotating the centroid tree with branch lengths

The approximate mean tree returned by our Centroid algorithm is a ranked tree and hence does not have branch lengths, only an ordering of internal nodes. Most applications, however, require time trees with branch lengths representing evolutionary event times. Because commonly used annotation methods, e.g. from the *TreeAnnotator* programme (Drummond and Rambaut 2007), change the ranking of a tree when annotating it, we cannot use them for our RNNI centroid approximation. We therefore introduce a new annotation method that does not change ranks of internal nodes in the summary tree. Instead of annotating branch lengths based on average heights of the respective clade (set of taxa below the node) as it is done by *TreeAnnotator*, we annotate with the average height of each rank, i.e. to calculate the height of the node of rank *i* we take the average of *i*’th *t*-coordinate (Gavryushkin and Drummond 2016) across the tree sample. For nonultrametric trees, we extend the *t*-space by extending leaf branches to create an ultrametric tree [similarly to Gavryushkin *et al.* (2018)]. This approach of annotating a ranked tree topology with branch lengths is a heuristic and we discuss its implications later in the paper (Supplementary Section S4).

### 2.6 The MCC summary tree heuristic

The *Maximum Clade Credibility* (MCC) tree is computed by the commonly used BEAST (Drummond and Rambaut 2007) utility programme *TreeAnnotator* in a two-step procedure. First the tree topology maximizing the posterior clade probability is chosen from the tree sample of the posterior distribution. This implies that an MCC tree contains the set of most probable clades from the sample of trees that is given. This tree is then annotated with clade ages, using one of four different methods. In this paper we consider the *common ancestor* annotation (Heled and Bouckaert 2013). The other three annotation techniques are discussed in the Supplementary Material (Supplementary Section S5.9).

### 2.7 Simulation study

To assess the performance of our approach we perform two sets of well-calibrated simulation studies in BEAST2 (Bouckaert *et al.* 2019). First, we simulate a ‘true’ tree and an alignment along this tree. Then BEAST2 is used to infer a posterior sample of trees from this alignment using the same model it was generated under, hence well calibrated. This type of simulation is commonly done because its outcome can be evaluated by comparing to the ground truth. We create two different simulations, one using the simple Jukes-Cantor (JC) (Jukes *et al.* 1969) model and one using the more complex Hasegawa–Kishino–Yano (HKY) (Hasegawa *et al.* 1985) model.

### 2.8 JC simulations

We generate a total of 504 different tree sets (i.e. posterior samples) for trees on a range of 50 up to 200 taxa. For each number *n* of taxa, we simulate approximately 50 different alignments and corresponding posterior samples, fewer for n≥100, as the run time of BEAST2 and our Centroid algorithm increases significantly with increasing number of taxa. The exact number of simulations for each set of taxa can be found in the Supplementary Section S5.4.

Tree and sequence simulations are done using R packages ape (Paradis and Schliep 2019) and phangorn (Schliep 2011). We use the JC model (Jukes *et al.* 1969) with a rate of 0.005 to generate sequence alignments of length 800. We run BEAST2 with this model specified, a chain length of 2.5 million samples, discarding first 500 000 as burn-in. Trees were sampled every 2000 iteration resulting in a tree set of 1001 trees. For larger analyses (taxa ≥100) we switched to 7 million samples (1 million burn-in) thinning to an output tree set of 2000 trees. A visual representation of the simulation workflow as well as the parameters used are displayed in Supplementary Fig. S13. Note that each simulation comprises one simulated alignment and one BEAST2 run on said alignment. To check convergence, we calculate ESS values using Tracer (Rambaut *et al.* 2018) and also use diagnostic tools from the RWTY package (Warren *et al.* 2017). Both confirm convergence using the commonly used ESS threshold of 200 and following the plot interpretation provided by Warren *et al.* (2017).

The major benefit of these more simplistic JC model is that we can easily compute the log-likelihood values of trees and we will use them throughout the later parts of this section.

### 2.9 HKY simulations

For the second set of more complex simulations, we used BEAST2 and its internal sequence generator. We generate 100 Yule trees with 100 taxa each and simulate alignments for each tree. For each alignment we run two independent BEAST2 MCMC chains, resulting in 200 sets of posterior samples.

The birth rate of the Yule (1925) process was fixed to 25.0. For the substitution model, we used the HKY+G model (Hasegawa *et al.* 1985). The shape parameter for the gamma distribution of site rates was modelled using a log-normal distribution, with a mean in log space of −1.0 and a standard deviation in log space of 0.5. The transition/transversion rate ratio (*κ*) also followed a log-normal distribution, with a mean in log space of 1.0 and a standard deviation in log space of 1.25. The nucleotide base frequencies were independently simulated for each replicate from a Dirichlet distribution with a concentration parameter array of [5.0, 5.0, 5.0, 5.0]. The length of the sequence alignments was 300 sites and the mutation rate was fixed at 1.0, so that divergence ages were in units of substitutions per site. The MCMC was run for 50 million iterations and sampled every 5000 iterations, resulting in a set of 50 000 trees in the posterior sample.

These simulations use a more complex model of sequence evolution and randomly distributed parameters. Moreover, by using shorter sequences the distribution within treespace becomes more realistic because we increase the uncertainty for the inference process. Overall, these tree distributions returned should be more complex than the ones returned by the JC simulations.

## 3 Results

In this section, we first present the results of our study comparing the approximate mean tree to the MCC tree, which is used as the default summary method in effectively all phylogenetic inference software packages. We then analyse the behaviour of the log-likelihood function during the execution of our approximation algorithm. The ‘as expected’ behaviour of the log-likelihood function in the RNNI treespace motivates our next investigation into the landscape of the log-likelihood function in different treespaces. We conclude the section by presenting applications of our method to three datasets in linguistics, phylogeography, and somatic evolution.

### 3.1 Mean trees in RNNI

In this section, we investigate how well our approach of approximating means of probability distributions over the RNNI treespace works in practice. Specifically, we establish various statistical and algorithmic properties of our method that are relevant for deploying our algorithm in practice. We start by comparing our approximate mean tree calculated by the centroid algorithm with the MCC tree, the current gold standard for summarizing samples of posterior distributions from a BEAST or BEAST2 analysis. In this comparison, we use the annotated centroid approximation (Supplementary Section S2.2.3) and the MCC tree with its common ancestor annotation (Supplementary Section S2.3).

**Figure 3.**
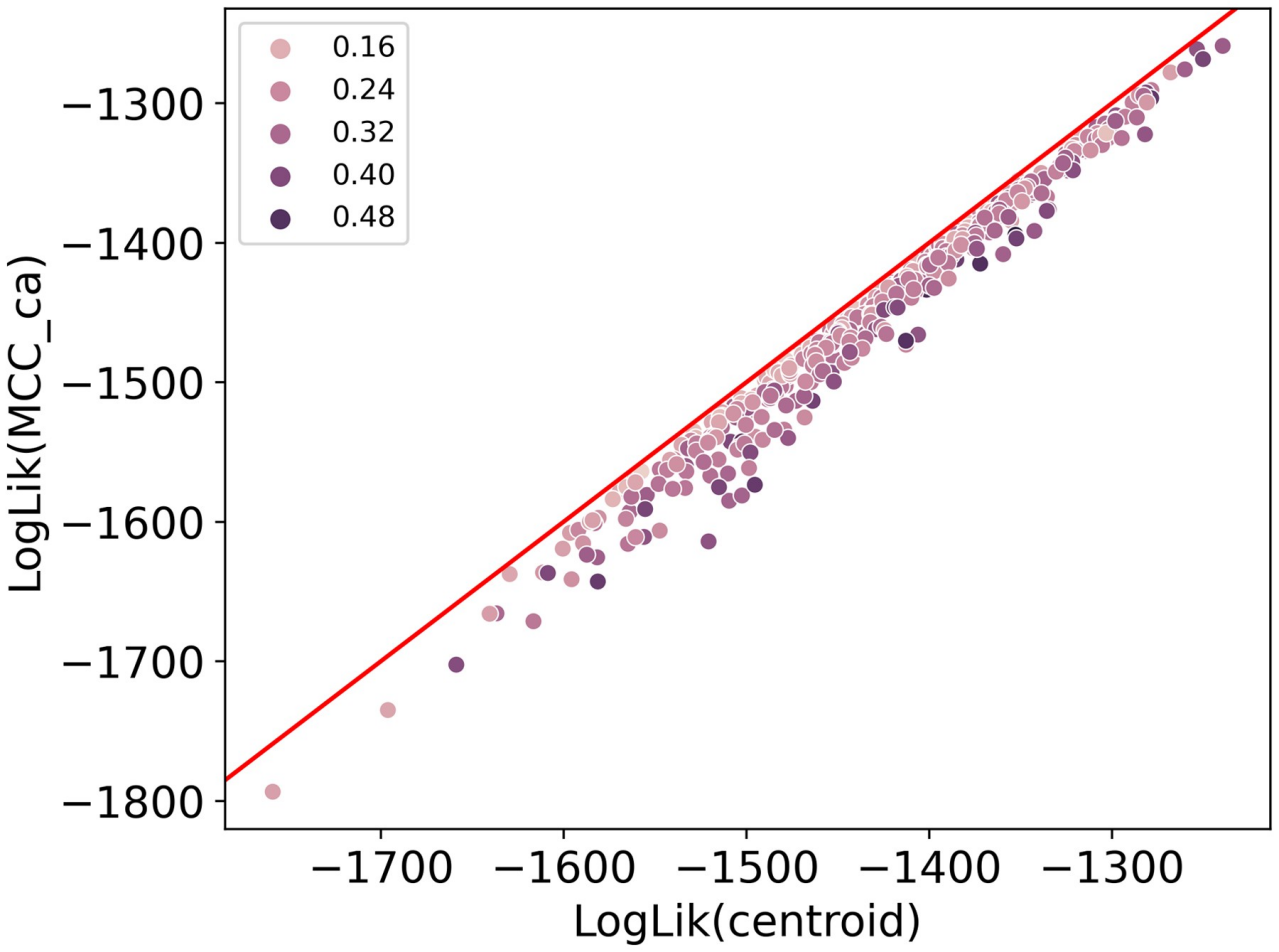
Comparing log-likelihood values of MCC trees with our centroid approximations for JC simulations. The data points are colour coded by their normalized RNNI distance (RNNI distance between the two summary trees divided by maximum possible RNNI distance). The red diagonal line shows the identity of the log-likelihood values.

Using the JC simulations as described in Supplementary Section S2.4, we compare the log-likelihood of our approximate mean tree with the log-likelihood of the MCC tree. The result is shown in Fig. 3, for the HKY simulations we cannot calculate the log-likelihood values of trees due to the randomized setup. The diversion off the diagonal (red) indicates that log-likelihood values are higher for the centroid approximation than for the MCC tree. The dots, each representing an individual simulation run, are coloured according to the RNNI distance between the two summary trees with darkness increasing with RNNI distance. Curiously, the darker dots are further away from the diagonal. We investigate this phenomenon in Supplementary Section S3.2.

A one-sided Mann–Whitney *U* test (Mann and Whitney 1947) [as implemented in Virtanen *et al.* (2020)] suggests that the distribution of log-likelihood values for centroid trees has a greater mean value than the one for MCC trees (*P*-value of 0.00013, indicating a significant difference in the means of the log-likelihood values). These results imply that our centroid approximation effectively provides a tree with higher likelihood than the MCC tree, making it a better summary of a tree sample.

**Figure 4.**
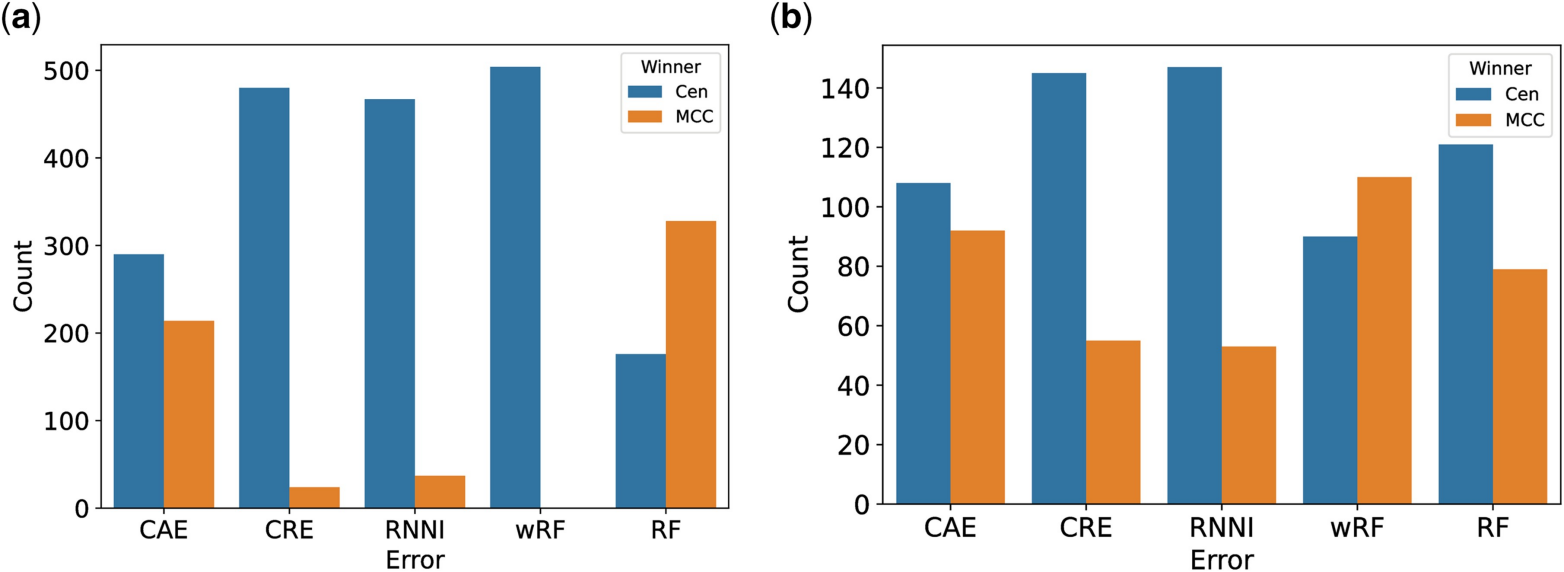
Comparison of the centroid approximation and MCC tree to the true tree using different evaluation measures: (a) JC and (b) HKY. The *y*-axis shows in how many simulations either MCC or the centroid approximation were closer to the true tree. The error measures are clade ages error (CAE), clades rank error (CRE), RNNI metric, weighted Robinson–Foulds (wRF), and regular Robinson–Foulds metric (RF).

In addition to this likelihood comparison, we consider a number of other measures to compare the centroid approximation to the MCC tree. For this, we distinguish JC-based simulation and HKY-based simulations. A summary of the results is presented in Fig. 4, where all measures compare the two summary trees (centroid and MCC) to the true tree used for the simulations. For the HKY simulation we extract tree sets of different sizes after discarding the first 1000 trees as burn-in. Within the main paper, we use the first 1000 trees after the discarded burnin, and equivalent results using the first 500, 2500, and 10 000 trees can be found in the Supplementary Material. More detailed figures displaying the exact values of error measures for both sets of simulations can be found in the Supplementary Sections S5.10 and S5.11.

The first two measures, clade age error (CAE) and clade rank error (CRE) (Heled and Bouckaert 2013), compare the height of clades in the respective summary tree to the true tree, and for both errors our mean tree approximation outperforms the MCC tree. The results for the clade rank error imply that the underlying ranked tree of the MCC tree is not as similar to the true tree as the ranked tree resulting from the Centroid algorithm. The result for the clade age error is surprising, as it suggests that our new method of annotating the ranked mean tree works well, even though it only takes heights of nodes into account regardless of the leaf labels below it. We also use the RNNI metric, where the approximate centroid tree is a more accurate approximation than the MCC tree.

We finally consider weighted and unweighted Robinson–Foulds distance between the summary trees and the true tree (Robinson and Foulds 1979, 1981). These metrics present the main difference between the two sets of simulations. While for our JC simulations the MCC tree is more often closer to the true three than to the centroid tree in terms of (unweighted) Robinson–Foulds distance, the opposite is true for our HKY simulations, where the centroid tree tends to be closer to the truth. The weighted Robinson–Foulds metric, however, consistently favours the centroid tree in the JC simulations, whereas in the HKY simulations, the MCC tree is more often closer to the true tree.

Overall, the results on the HKY simulations are less one sided but with the centroid tree outperforming the MCC tree on average in almost all measures across the different simulation setups.

We additionally analysed smaller datasets with 8–50 taxa, which show similar results (see Supplementary Fig. S21). Only for trees with fewer than 20 taxa, the centroid approximation and the MCC tree were mostly identical, which indicates that when the tree sample is well converged both summary methods are able to approximate the mean tree well. However, the difference of the two summary trees increases significantly when the number of taxa increases. This can be thought of as the sample being sufficient for these smaller taxa simulations, due to the MCC tree being a sampled tree, while for larger problems the sample is insufficient and a geometric mean approach is superior.

To disentangle the effect of our branch length annotation method (Supplementary Section S2.2.3) from our estimation of the ranked tree topology, we investigated the impact of changing the branch length annotation method. Specifically, we compared log-likelihood values of the MCC tree topology annotated with the three default methods provided in TreeAnnotator with our newly presented method. The comparison demonstrates that there is no significant difference of log-likelihood values in the MCC trees returned by either of these branch length annotations. These results are visualized in the Supplementary Material (Supplementary Figs S16, S17, and S22). A detailed comparison for each of the error measures from Fig. 4 can also be found in the Supplementary Material (Supplementary Fig. S18).

Our method is implemented to run in practical time and is unrestricted in its exploration of the RNNI treespace to find a good candidate tree (see the Supplementary Section S5.7 for details), improving over existing geometric mean approaches that suffer from long runtime and only explore the respective treespace partially, often restricted to a small set of input trees (McMorris and Steel 1994, Heled and Bouckaert 2013).

### 3.2 Likelihood and posterior distributions over the RNNI treespace

**Figure 5.**
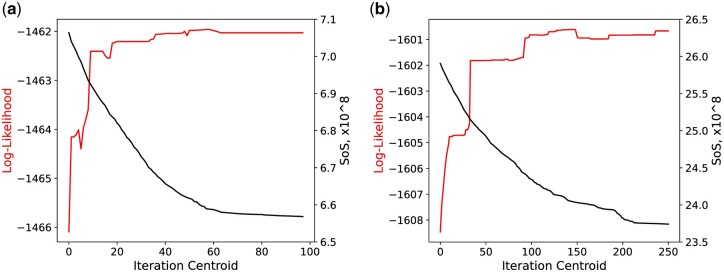
Log-likelihood and SoS values for Centroid runs on a simulated dataset with (a) 61 taxa and (b) 100 taxa on the right.

In this section, we further investigate the relationship between the geometry of the RNNI treespace and the likelihood function. Specifically, we are looking into the changes of the likelihood function during execution of the Centroid algorithm. Recall that at every iteration of the algorithm the SoS value from the running tree to the tree sample decreases. The result is depicted in Fig. 5, where we plot the log-likelihood value against the SoS value over the iterations of the Centroid algorithm (Algorithm 1). We can see that while the algorithm minimizes the SoS value of the approximate mean tree, the log-likelihood value increases. Hence, the Centroid algorithm finds a tree with higher and higher log-likelihood while only using the geometry of the RNNI treespace. This connection between the geometry of the space and the behaviour of likelihood functions indicates the appropriateness of this treespace for statistical analyses of phylogenetic time trees.

**Figure 6.**
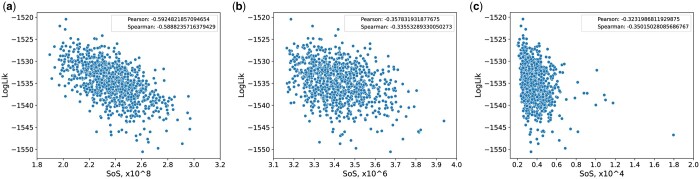
Log-likelihood values against SoS values for a set of trees from a simulation on 50 taxa in different treespaces: (a) RNNI, (b) RF, and (c) BHV. The top right legend contains the Spearman and Pearson correlation coefficient. The average correlation values are roughly −0.59 for RNNI, −0.34 for Robinson–Foulds, and −0.34 for BHV. See Supplementary Material for correlation coefficients among all simulated datasets within the RNNI treespace.

To further investigate the relationship between the geometry of the RNNI treespace and the likelihood function, we investigate the correlation between the SoS and log-likelihood value of a tree. We found a negative correlation with both the Pearson and the Spearman tests [as implemented in Virtanen *et al.* (2020)] ranging between −0.4 and −0.6 among our simulated datasets, see Supplementary Material for a visualization of these values. In Fig. 6, we compare the SoS and log-likelihood values for every tree within one simulated dataset on 50 taxa for three different treespaces. According to this negative correlation we have found another indication that the RNNI space is consistent with statistical intuition, i.e. the tree at the peak of the log-likelihood distribution should also be a minimum for the SoS values. In addition, Fig. 6 displays a comparison of this property on an example set of trees in the Robinson–Foulds and BHV treespace.

Finally, we investigate how continuous the log-likelihood function is in different treespaces. The intuition here is that local changes in a treespace should correspond to local changes in likelihood and posterior probabilities of the corresponding trees. A degree of continuity (or ‘smoothness’) is a desirable property for the commonly deployed hill climbing tree search algorithms in both Bayesian and maximum likelihood frameworks.

**Figure 7.**
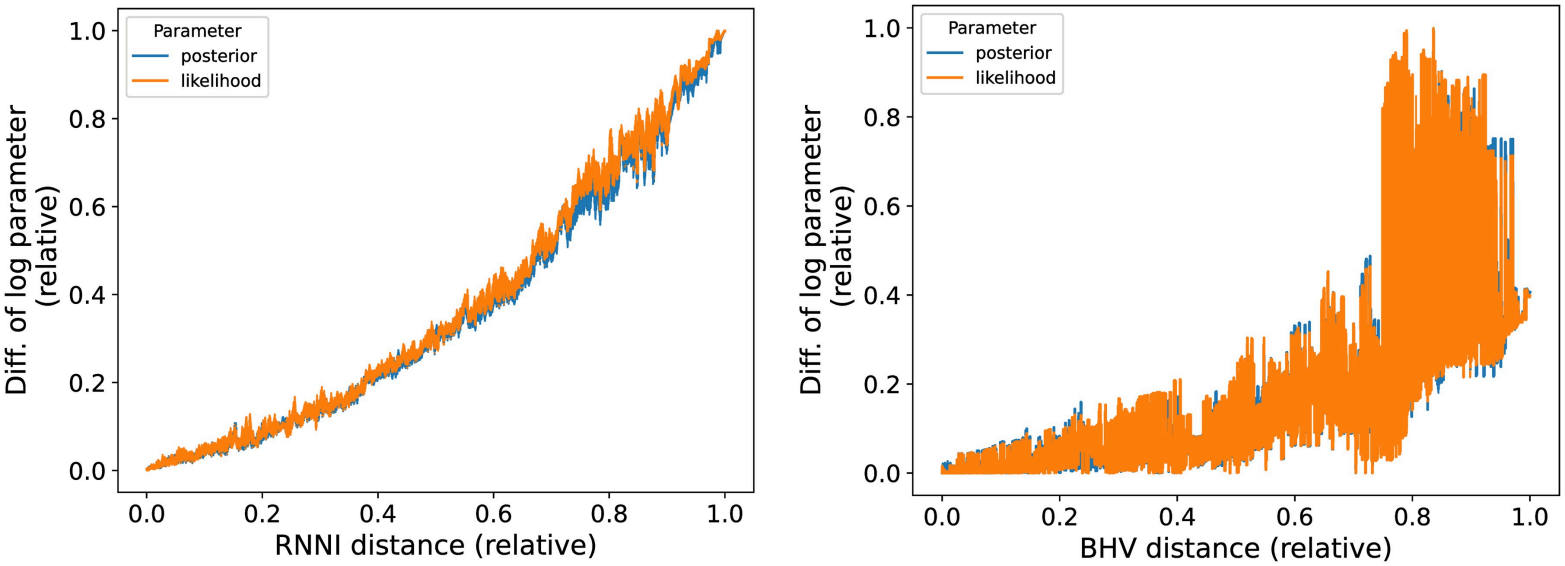
Smoothness of probability distributions over the RNNI and the BHV treespace. The plot compare the two distances between every pair of trees from an MCMC chain with the difference between their log-likelihood and posterior probabilities normalized by largest observed values.

Using 1000 tree samples obtained from the beginning of an MCMC analysis of simulated sequence data (see Supplementary Section S5.13 for details), we compare the RNNI distance between every pair of such trees with the difference between their log-likelihood and posterior probabilities (both distances and probabilities are normalized by their corresponding largest observed values). We also repeat this comparison for the BHV, Kendall–Colijn (Kendall and Colijn 2016), and Robinson–Foulds metrics. Note that these metrics are designed for standard phylogenetic (rather than time) trees, and it has been shown (Gavryushkin and Drummond 2016) that adapting standard metrics to time trees is nontrivial. We found that both log-likelihood and posterior probabilities are ‘smooth’ in the RNNI and Robinson–Foulds spaces but not in BHV and Kendall–Colijn. The result for BHV and the RNNI treespace is shown in Fig. 7, and results for Robinson–Foulds and Kendall–Colijn metrics can be found in Supplementary Fig. S24.

### 3.3 Real data examples

In this section, we apply our method to three problems in applied phylogenetics. These include natural language evolution (Kolipakam *et al.* 2018), molecular evolution (Kitson *et al.* 2018), and cancer evolution (Alves *et al.* 2019). In all three cases our method provided additional insights into (if not alternative hypotheses about) the corresponding evolutionary processes, demonstrating the value of our approach in practice.

### 3.4 Dravidian languages

Our first application is in evolutionary linguistics. Specifically, we analyse the Dravidian language dataset published by Kolipakam *et al.* (2018). The Dravidian languages are a diverse family of languages spoken by about 250 million people predominantly across southern and central India. The original study aims to provide a time-depth estimation of divergence between languages and a reconstructed evolutionary history of the language family. These results contribute to a deeper understanding of the historical development and diversification of the Dravidian language family, a topic of ongoing research with many unanswered questions.

**Figure 8.**
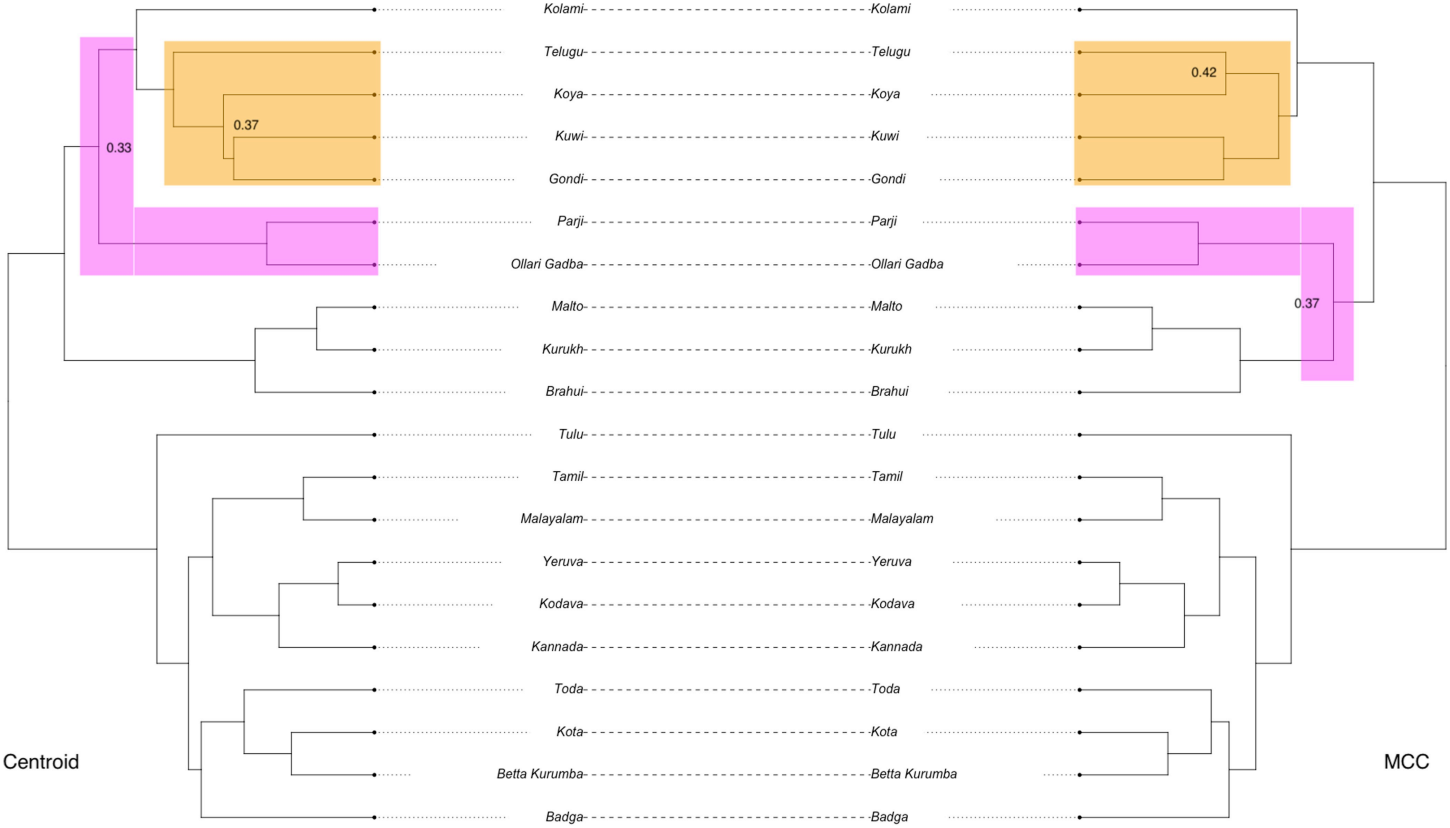
Comparing the MCC tree (right) to the centroid approximation (left). Highlighted clades have a changed topology or position in the centroid tree versus the MCC tree.

For our analyses we took a BEAST2 sample of trees generated in the original study (Kolipakam *et al.* 2018), applied our method, and compared the result to the published (MCC) tree. As it can be observed in Fig. 8 the topology of our (centroid) tree is slightly different to the MCC tree (highlighted in yellow in the figure). In particular, our tree splits the sister relationship of the two languages Telugu and Koya placing Koya as a descendant of Telugu. As this clade of the South II languages is of specific interest in Kolipakam *et al.* (2018), where further investigation into this part of the tree is suggested, our result can serve as an argument in favour of this particular placement of the two languages.

The other difference between the two trees (highlighted in pink in Fig. 8) is the degree of separation between the three Central languages (Parji, Ollari Gadba, and Kolami). Specifically, the two languages Parji and Ollari Gadba are closer to the Kolami language in the centroid tree whereas these form a distinct clade with the three North languages (Kurukh, Malto, and Brahui) in the MCC tree. This difference is consistent with the proximity network provided in Fig. 2 in Kolipakam *et al.* (2018).

These discrepancies may be caused by convergence issues or a multimodal posterior distribution, as indicated by very similar posterior support values seen in Fig. 8. Note that the MCC is actively maximizing posterior support and therefore will always have higher or equal support than the centroid topology. Thus, in the context of data and discussion presented in Kolipakam *et al.* (2018), we conclude that the centroid topology provides valuable insights into the data and evolutionary process under consideration.

### 3.5 Weevils

Our second application falls within the study of molecular evolution, specifically investigating in situ speciation on the islands Mauritius and Reunion of Cratopine weevils. The study applies phylogenetic and biogeographic methods to analyse genetic data from multiple weevil species and reconstruct their evolutionary relationships and dispersal patterns. The results of the study provide new insights into the processes of community assembly and diversification in a species-rich island radiation, and highlight the role of historical and ecological factors in shaping the evolution of these weevils. Here, we are interested in the specific differences of topologies between the MCC and our centroid tree and their implications for the dataset.

As noted by Kitson *et al.* (2018) the position of the *C.nigrogranatus* in the phylogeny is unresolved. We find that its position is indeed different for the two trees, see Supplementary Fig. S25. Moreover, we find that the position of one of the Rodrigues clades containing *C.virescens* and *C.viridipunctatus* has changed its position within the larger subtree. It does in fact not move any closer to the second clade thought to colonize Rodrigues keeping the question of multiple colonizations of Rodrigues open. This clade is now located in the middle of the clade 2b [see Fig. 2 in Kitson *et al.* (2018)], suggesting a different lineage for these species and the respective colonization process of the islands.

### 3.6 Cancer evolution

Our third application is within the domain of cancer evolution, specifically the evolution and spread of colorectal cancer cells within a patient. Alves *et al.* (2019) presented tumour clones computationally inferred from whole-exome sequencing data and analysed the history of cancer cells evolving and spreading to different organs over time. The results from this study provide important insights into evolutionary dynamics of cancer within a single patient by identifying tumour demographics and colonization patterns in a defined time-frame. Here, we repeat the original analysis using our centroid method and compare the result with the published (MCC) tree.

**Figure 9.**
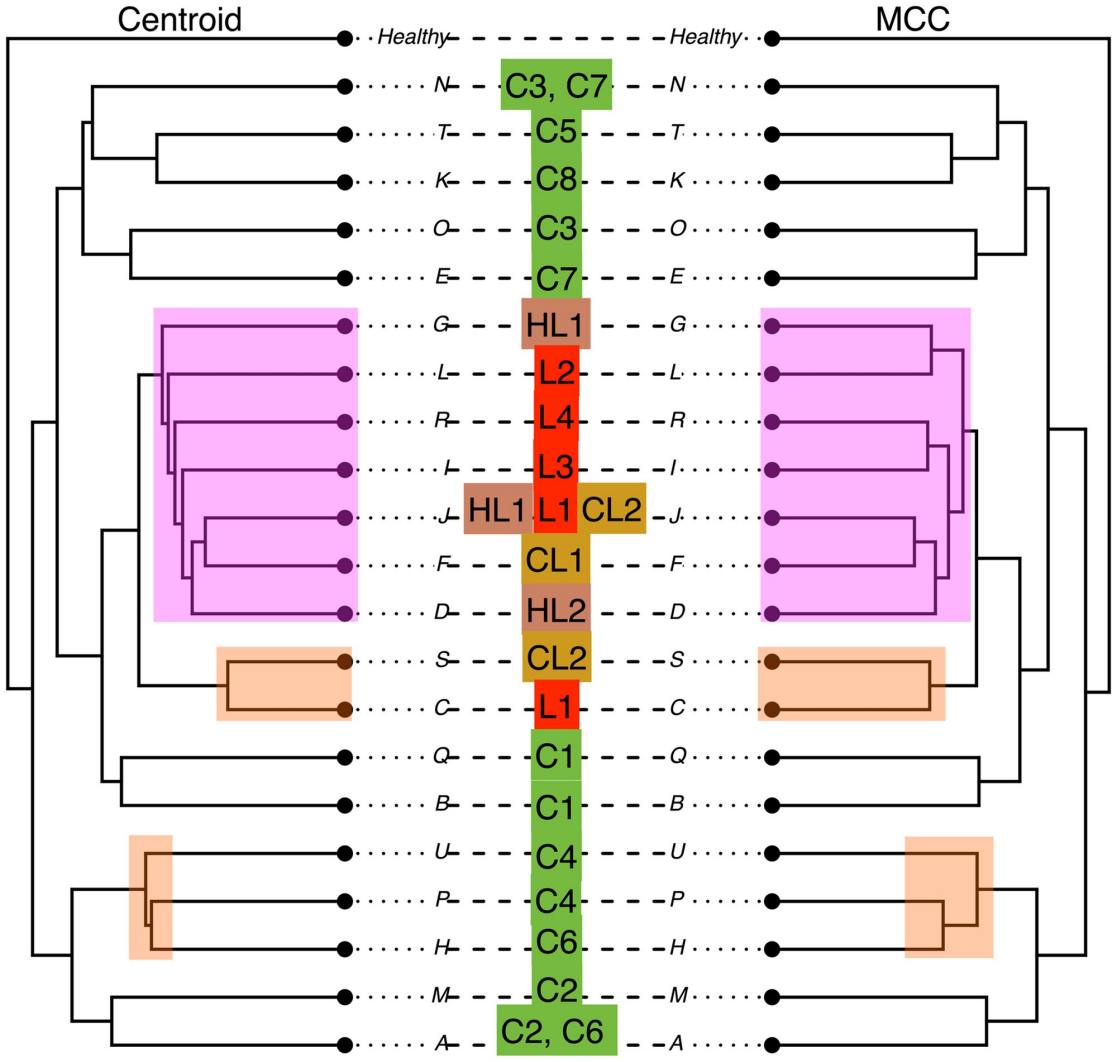
Highlighted differences in the two trees for the analysis of cancer data.

The main difference between the two trees is a change of the subtree topology corresponding to the liver, colonic lymph node, and hepatic lymph node metastases clade (taxa D, F, J, I, R, L, G in Fig. 9). In the MCC tree the corresponding subtree is a fully balanced topology, in which the liver cells are grouped with colonic and hepatic lymph node cells as cherries in the tree. In the centroid tree on the other side, this clade is resolved as a caterpillar topology where all the liver cells are grouped as single leafs one after another. Because the tree shape can be informative of the type of evolutionary process (Mooers and Heard 1997, Schwarz *et al.* 2015) and e.g. can be used to distinguish among different epidemiological scenarios, the difference between the MCC and centroid tree shapes can point at different evolutionary processes driving cancer development in this clade. In Fig. 9, we also highlight other less significant differences in branch lengths and timing of nodes.

## 4 Discussion

The RNNI treespace has been developed specifically for phylogenetic time trees and it is the only treespace, to the best of our knowledge, that is based on local tree rearrangement operations with efficiently computable distances and paths. In this paper, we study the question of whether this property enables the RNNI space to be suitable for developing probability theory and statistics over the space of phylogenetic time trees. We show a number of promising results that collectively suggest that the answer to this question is ‘yes, but a significant amount of work is yet to be done’. For example, we are able to demonstrate a strong connection between RNNI as a metric space and log-likelihood as a probability distribution over this space. This connection came in the form of a smoothness property of the log-likelihood functions over the RNNI space, which we believe is a desirable property of a treespace. Further, we developed an algorithm, Centroid, for approximating means of probability distributions in this treespace. As a practical consequence of this algorithm, we demonstrated its excellent performance at summarizing samples from posterior distributions in the full Bayesian framework. This result has concrete consequences for data analysis, and we demonstrated this by applying our algorithm in three areas including natural language, molecular, and cancer evolution. In all three cases, the Centroid tree was different from the MCC tree reported in the original study. Although in all cases our summary tree was compatible with the discussions in the respective papers, it supported some of the alternative hypotheses the authors considered originally. So we conclude that our method has immediate practical utility.

Having scratched the surface of what we believe can be achieved with a local rearrangement based treespace with efficiently computable distances and paths, we finish this paper by outlining some of the possible ways forward. First, we summarize the properties of the RNNI treespace that we believe are fundamental for a treespace to possess in order to be suitable for statistical analyses. We intentionally leave the statements of these properties mathematically informal as their formalization would inevitably leave some potentially fruitful approaches to developing statistics over treespaces out, which is something we intentionally avoid. For example, we would like to avoid requiring the treespace to be a metric space.

1. The treespace is local, i.e. the notion of a (local *δ*-) neighbourhood is well-defined and all trees within a neighbourhood are ‘similar’; computationally, neighbourhoods need to be efficiently computable/samplable. For RNNI this is enabled by tree rearrangement operations.
2. The notion of ‘betweenness’ is well-defined, i.e. it is possible to find a weighted average between two (or more) trees, and the average tree is ‘similar’ to the trees that are being averaged; computationally, these need to be efficiently computable or approximated. For RNNI this is enabled by shortest paths satisfying the cluster property and the property that all trees on shortest paths are fully resolved.
3. Probability distributions (of importance) over the treespace are ‘smooth’, i.e. similar trees have similar probabilities; computationally, the likelihood functions need to be efficiently samplable. For RNNI this is enabled by standard phylogenetic likelihood functions being efficiently computable and local with respect to tree rearrangement operations.

This list is certainly not exhaustive and our main reason for providing it here is to encourage a discussion of desirable properties a treespace should possess depending on the use case. We are hopeful that this discussion would enable a more purposeful treespace design and would guide phylogenetic practitioners whenever a choice needs to be made regarding what treespace to use.

Second, we provide a list of mathematical problems that we believe would be important to solve.

1. Given a set *S* of *m* trees in RNNI such that *m* > *n* (the number of leaves):
  - How many mean trees can *S* have?
  - Can a mean tree of *S* be computed in polynomial in *m* time?
  - Can all mean trees of *S* be computed in polynomial in *m* time, assuming the number of mean trees of *S* is polynomial in *m*?
    Related to this is the question of whether the running time of the Centroid algorithm can be improved, i.e. whether there exists a faster algorithm for approximating mean trees in the RNNI space. Although the running time of the Centroid algorithm for big trees is still significantly higher than that of the MCC algorithm, there is nothing to suggest that our implementation is optimal.
    We, in fact, have carried out several computational experiments aimed at understanding the number of mean trees, as well as scenarios when the Centroid algorithm fails to find a mean tree and the impact of the starting tree on the algorithm’s performance. These are presented in Supplementary Section S5.1.
2. Given the mean tree in the RNNI space:
  - Is the branch length annotation we use for ranked trees statistically consistent?
  - Is there a better way to annotate branch lengths?

Although potentially statistically inconsistent, our annotation method works well in practice. Either way, we have little doubt that our method can and should be improved.

## Supplementary Material

btae514_Supplementary_Data

## Data Availability

The simulated data used in this article can be reproduced using the instructions provided in the article and in the online supplementary material. The real datasets can be accessed through the original references provided.
