# Supplementary material for: Estimating the mean in the space of ranked phylogenetic trees
Source: Bioinformatics. 2024 Aug 23;40(8):btae514. doi: 10.1093/bioinformatics/btae514 (PMC11364146; doi:10.1093/bioinformatics/btae514)
Supplement: btae514_Supplementary_Data [file btae514_supplementary_data.pdf]

Supplement

Centroid properties

Finding global optima

To conduct a test of whether or not our algorithm is able to recover global optima as presented in Figure 10, we implemented the following approach:

- Generate all trees of the treespace (up to 7 taxa)
- Generate a random tree set in this space
- Compute the SoS for all trees and save the global optimum of SoS
- Start the CENTROID[2] algorithm from every tree in the tree set
- Combine all solutions and choose the best one (minimal SoS)
- Check if it is a global optimum

		# trees				
		5	10	25	50	100
# taxa	4	0/50 (94)	0/50 (92)	0/50 (90)	0/50 (86)	0/50 (88)
	5	0/50 (92)	0/50 (89)	0/50 (86)	0/50 (85)	0/50 (82)
	6	0/10 (94)	0/10 (79)	0/10 (74)	NA	NA
	7	0/5 (72)	NA	NA	NA	NA

Fig. 10: Displays the results of tests to see if the CENTROID algorithm finds a globally optimal solution. The row index shows the number of taxa and the column index the size of the randomly generated tree set. An entry x/y (z) displays the number of times x the algorithm did not find a globally optimal solution out of y different randomly generated tree sets. The value (z) displays the percentage of trees in the tree sets that when used as a starting tree found a global optimum. NA are not conducted tests because of the increasing time complexity.

We conclude that the algorithm is likely to always find a global optimal solution when the starting tree is chosen correctly. However, starting the algorithm from every tree in the set and also following all paths that decrease the objective function, i.e. the SoS value, already takes a long time in these small treespaces and tests on 6 and 7 taxa were only conducted on very small tree sets (see NA in Figure 10).

How many global optima are there?

We further investigate these globally optimal solutions, specifically in the context of how many such optima exist and also whether or not these optima form a connected subgraph in the treespace. The findings presented in Figure 11 lead us to formulate the following conjecture

Conjecture 5.1. *For a sufficiently large tree set there exists a unique centroid tree.*

Starting Tree algorithm

Pseudocode for the variation of Strums algorithm (Sturm, 2003) adapted to the RNNI treespace. For geodesics we use the shortest path within the graph that is computed by the findpath algorithm (Collienne et al., 2021; Collienne and Gavryushkin, 2021).

[2] For this result we use a minor modification to the CENTROID algorithm that follows every possible path, i.e. every neighbour with lower SoS is pursued by the next iteration of the algorithm.

Algorithm 2 STARTING TREE

```
procedure STARTING TREE(𝒯)
    FM ← 𝒯.pop(random)
    d ← 2
    while 𝒯 not ∅ do:
        t ← 𝒯.pop(random)
        𝒫 ← path(t, FM)
        FM ← 𝒫[⌊length(𝒫)/d⌋]
        d ← d + 1
    end while
    return FM
end procedure
```

In addition we run some tests on smaller dataset to compare the output of this algorithm with any tree that could be picked from the original tree set. It turned out that a starting tree computed by this algorithm is consistently below the expected SoS value one would get by picking a tree in the given set of trees. This result is consistent with geometrical intuition in a treespace.

Error Measures

Most of these definitions for error measures can be found in (Heled and Bouckaert, 2013).

Clade ages error - CAE

The *clade ages error* CAE, the sum of differences between clade ages, for a tree T and a reference tree T_R is defined as

$$\mathrm{CAE}(T, T_R) = \sum_{\text{clade } c \in T_R} |\mathrm{height}_T(c) - \mathrm{height}_{T_R}(c)|.$$

For a clade c in T_R that is not in a tree T we define $\mathrm{height}_T(c) = \mathrm{height}_T(mrca(c))$. It is important to note that this error is not symmetrical.

Clades missed error - CME

The *clades missed error* CME counts the number of clades present in the reference tree T_R which are not in T:

$$\mathrm{CME}(T, T_R) = |\{\text{clade } c \ \in T_R : \text{clade} c \notin T\}.$$

This error is exactly half of the RF distance (Heled and Bouckaert, 2013).

Clades called error - CCE

The *clades called error* CCE scores $+1$ for correct clades and -1 for incorrect clades in a tree T, compared to a reference tree T_R:

$$\mathrm{CCE}(T, T_R) = |\{c \in T : c \in T_R\}| - |\{c \in T : c \notin T_R\}|$$

Clade rank error - CRE

The *clade rank error* CRE is a deviation of the clade ages error CAE. It is the sum of differences between clade ranks, therefore this error measure is specific for ranked phylogenetic trees:

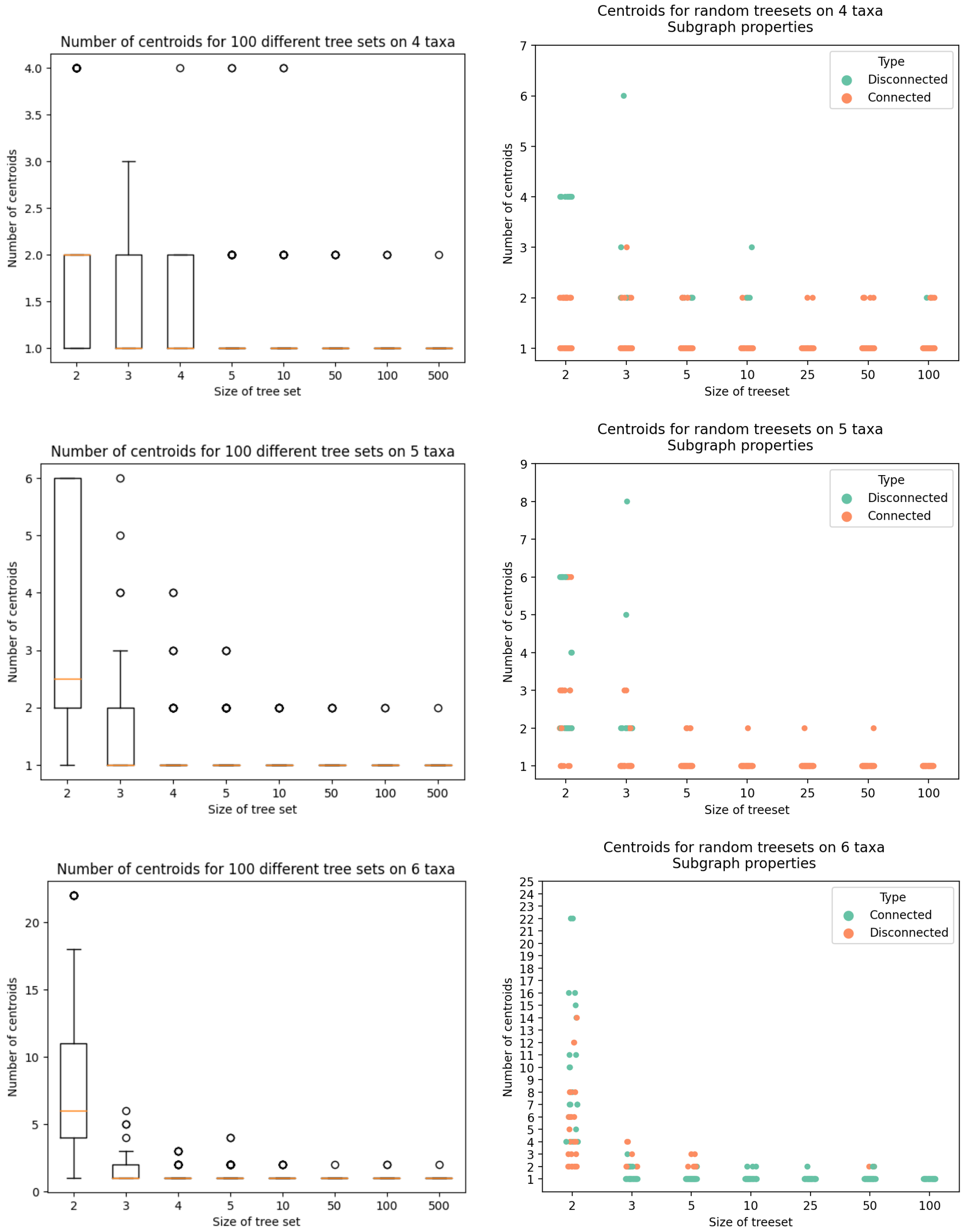

Fig. 11: Evaluating number of centroids and their connectedness on small taxa RNNI treespaces. Left column plots are for 100 randomly generated tree sets of different sizes and the right column plots are from 50 randomly generated tree sets.

$$\mathrm{CRE}(T, T_R) = \sum_{clade\ \ c\ \ \in T_R} |rank_T(c) - rank_{T_R}(c)|.$$

Analogue to the CAE, the rank of a clade c that is not in a tree is defined as the rank of the most recent common ancestor of that clade. This error is also not symmetrical.

Tree metrics

The considered tree metrics and their used abbreviations.

- Robinson-Foulds – RF
- weighted Robinson-Foulds – wRF
- Path difference – PD
- weighted path difference – wPD
- Branch Score difference – KF
- RNNI distance – RNNI

Log Likelihood value

For the log-likelihood value we use the *pml* function from the R package *Phangorn* (Schliep, 2011).

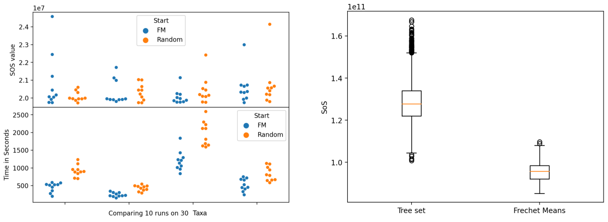

(a) (b)

Fig. 12: (a) Comparing 4x10 different executions of the Centroid algorithm with a random starting tree from the set vs. a tree computed by the Starting Tree algorithm (FM in the plot) on a dataset with 30 taxa. The top half displays the SoS value of the returned tree of the algorithm and the bottom shows the runtime of a Centroidcomputation using either of the starting trees. (b) Comparing the SoS value of all samples in a set of trees versus the SoS value of starting trees computed by the Starting Tree algorithm on a dataset with 188 taxa. Due to the random choice of trees in the Starting Tree algorithm there is some variance in the returned tree.

Simulation Study - setup

In Figure 13 the setup of the Jukes Cantor based simulations is visualized. For these simulations we use the R packages *ape* (Paradis and Schliep, 2019), *phangorn* (Schliep, 2011), *babette* (Bilderbeek and Etienne, 2018), and *RWTY* (Warren et al., 2017).

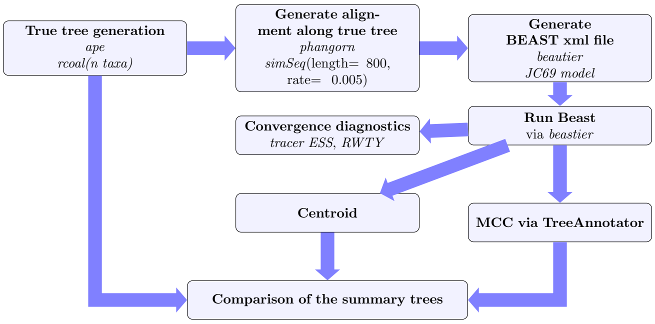

Fig. 13: Flow chart of the JC simulation studies

Simulation Study - number of simulations

The following table Table 1 shows the actual number of simulations that were conducted. The reason for the "weird" uneven numbers are solely for the ease of implementation and due to increasing complexity and time consumption for higher numbers of taxa.

n taxa	50	51	55	60	65	70	75	80	90	100	120	125	140	150	175	200	Total
k simulations	50	10	50	50	50	50	50	51	50	20	8	13	15	15	12	10	504

Table 1. Overview of the data used in the following comparison

Simulation Study - Convergence Checks

We checked for convergence of our simulated data sets with the current state of the art tools. For this we checked that the ESS values are all well above 200 with the Tracer tool. Additionally we used the R package RWTY to check different diagnostics.

Conversion from branch lengths to ranking

Because the trees returned by a BEAST analysis are time trees the real times have to be converted to the discrete ranks of the RNNI trees. There are different ways of calculating the corresponding ranked tree. For these results we used a top down approach for conversion where the ranks are inferred by the corresponding t-space coordinates (Gavryushkin and Drummond, 2016), similar to how we annotate a tree with branch lengths subsubsection 2.2.3.

Running time of Centroid algorithm

Because of the problems with long running times of (Heled and Bouckaert, 2013; McMorris and Steel, 1994) previous attempts at implementing a geometric mean based method we evaluate our implemented approximation. We found that due to our use of the greedy path heuristic in combination with the efficiently computable RNNI distance that the algorithm is running in reasonable time. Unlike the algorithm presented in (Heled and Bouckaert, 2013) (named minimum distance tree) our method is also not restricted on a subset of trees and able to explore all of treespace. These tests are performed using 8 or 16 threads across two eight-core (16 SMT threads) Intel(R) Xeon(R) Gold 6244 CPUs in a NUMA system and a summary is provided in Figure 14. In Figure 14 we present running times for the Jukes Cantor based simulations and in Table 2 we present runtimes on the more complex HKY simulations.

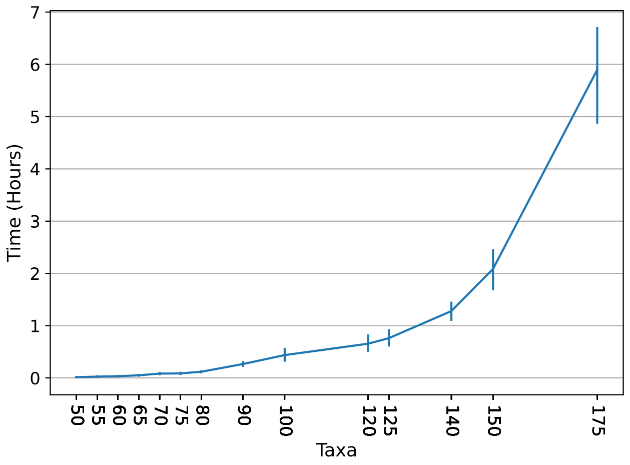

Fig. 14: Evaluating the running time of the Centroid algorithm as implemented in `https://github.com/bioDS/Centroid-Code` using samples of trees with different number of taxa (x axis). For up to 100 taxa the sets contain 1001 trees and the larger simulations contain 2001 trees, these originate from the Jukes Cantor simulations described in the main paper. The error bars originate from 10 individual runs of the algorithm on data of the same size.

Table 2. Runtime of our implementation on different sized datasets with 1000 trees using 16 cores.

Number of taxa	Time Unit	Fastest	Average	Slowest	Repetitions
40	Seconds	1.73	4.03	18.88	90
80	Seconds	19.60	46.52	83.99	80
100	Minutes	3.35	4.67	6.24	80
200	Hours	1.32	1.75	2.08	5

Correlation of SoS and log-likelihood

As stated in the main paper we found negative correlation between the log-likelihood and the SoS value of trees. In Figure 15 we visualize the correlation coefficients among all datasets separated by the number of taxa.

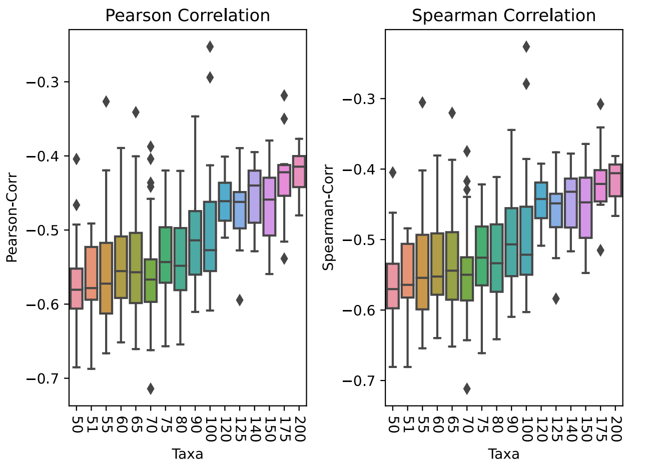

Fig. 15: Pearson and Spearman correlation coefficients for all simulated datasets, separated by number of taxa.

Different MCC branch length annotations

The *TreeAnnotator* program provides three different branch length annotation options. The first option uses the ages as they are in the tree picked from the posterior sample. The Second and third option compute mean and median clade ages, respectively, for each clade based on their age in all trees from the posterior sample. Both of these annotations can result in negative branch lengths, because the average height of one clade in the MCC tree may be higher than the average height of its parent, see `https://www.beast2.org/summarizing-posterior-trees/`. The fourth option computes the average most recent common ancestor time for each clade, therefore avoiding negative branches being annotated (Heled and Bouckaert, 2013).

We compare the three different tree annotations for the MCC tree (we do not consider the method of keeping the branch lengths from the picked tree here because we think it is vital to use all the information of an analysis and not just pick one specific tree) to choose the best one for the comparison with our new method. The three differently annotated MCC trees are compared based on their log-likelihood values, as displayed in Figure 17. The plot shows slightly worse log-likelihood values for the common ancestor option than for median and mean branch length annotation. We conduct a Mann-Whitney-U test (Mann and Whitney, 1947) on the log-likelihood values and found no significant difference in the log-likelihood values of these three annotations. To investigate whether our method, presented in subsubsection 2.2.3, of annotating branch lengths improves the MCC summary tree, we took the ranked tree topology produced by the MCC, annotated it using our method, and compared the result with the mean MCC branch length annotation as implemented in TreeAnnotator. We found no significant difference between the log-likelihood of these two trees (visualized in Figure 16) and therefore, we only compare the centroid approximation to a MCC tree using either of the three already available branch length annotations. However, as both mean and median branch length annotation result in negative branch lengths in some of our simulations, we compare our centroid approximation with the common ancestor annotation of the MCC tree.

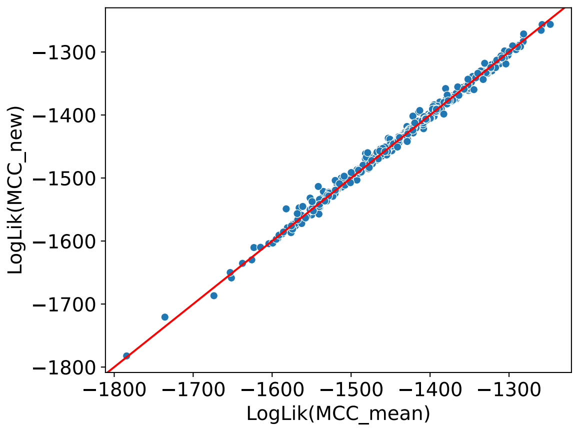

Fig. 16: Comparing the log-likelihood values of the mean MCC branch length annotation versus our presented way of annotating a ranked tree (subsubsection 2.2.3). This plot displays slightly more deviation of the red diagonal line than present in any of the three comparisons of Figure 17.

Detailed scatter plots for summary comparison of JC simulations

The main paper shows a summary of the following outcomes (Figure 18) of comparing the MCC and our centroid approximation in a histogram plot (Figure 4). Here we add the path difference metric (Steel and Penny, 1993) to the list of error measures. For it, the MCC always outperforms the centroid tree, however the values of the distance are orders of magnitude different especially for simulations with more taxa. We recognize that this is a strange behaviour of the metric and we are unaware as to why this is the case leaving us without a conclusion on this result.

Detailed scatter plots for summary comparison and different subsets for HKY simulations

In Figure 19 we display the values of the error measures used to plot a summary histogram plot. In Figure 20 we display the summary histograms for all different sized subsets. There is no notable difference or trend to be observed with less or more trees in the sample used.

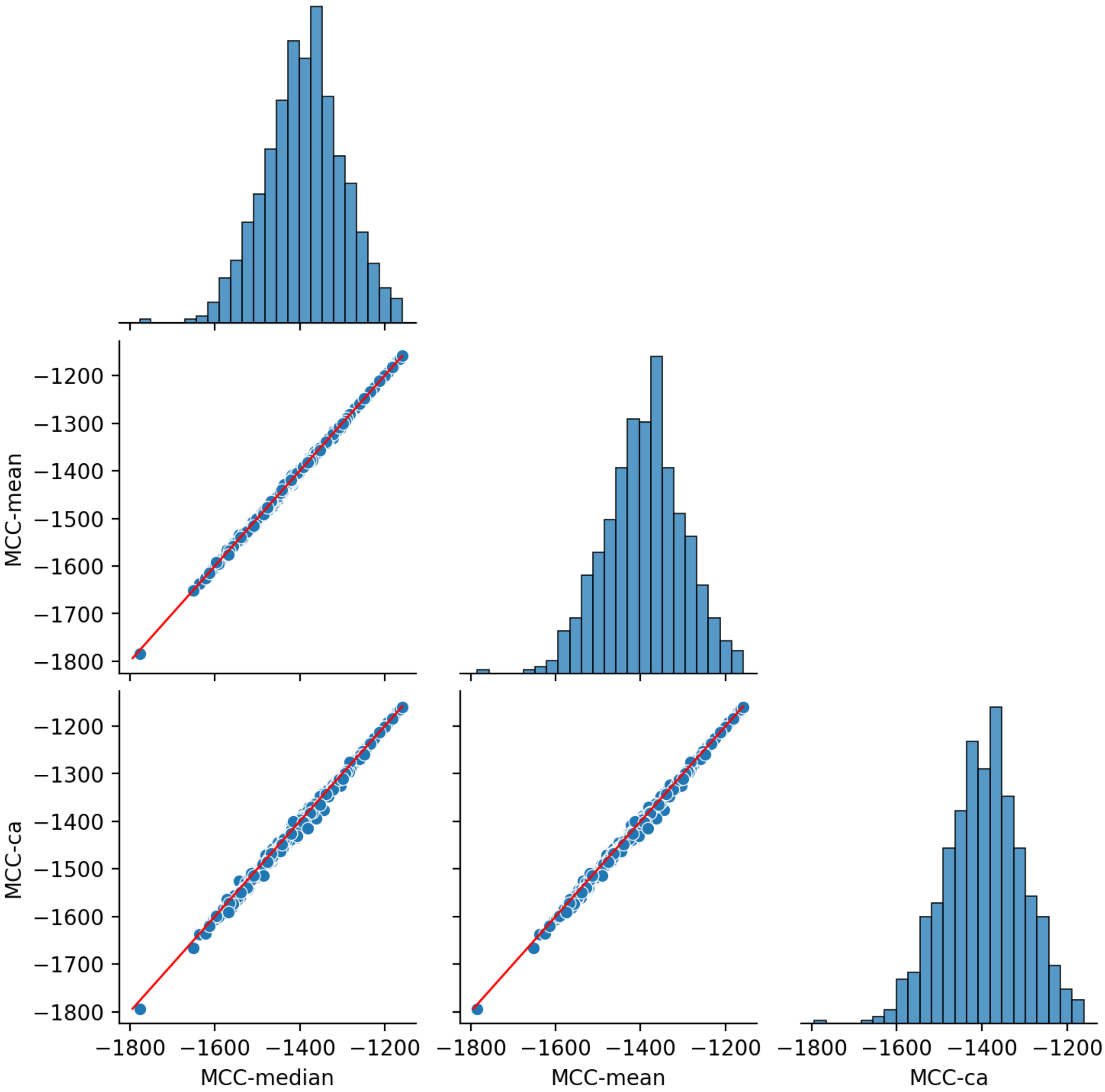

Fig. 17: Comparing the log-likelihood values for different MCC branch length annotations. Deviation of the red diagonal line in the scatter plots indicate which tree annotation achieves higher log-likelihood values. The histograms show a distribution of the log-likelihood values for each of the annotations.

More comparison plots

As stated in the main paper we only focused on simulations with more or equal to 50 taxa. The plot Figure 21 shows a comparison when datasets with fewer taxa are included. In Figure 22 a comparison using the median branch length annotation for the MCC tree can be seen. For that comparison the bars are divided by whether or not the MCC tree considered contains negative branch lengths or not. The plot shows that a vast majority of the MCC annotations contain negative lengths.

Smoothness analysis

We analyse the smoothness property for the RNNI, BHV, Kendall-Colijn, and Robinson-Foulds spaces by considering likelihood and posterior probabilities of a set of trees obtained from the following data: we simulate an alignment of 800bp for 100 taxa and run a

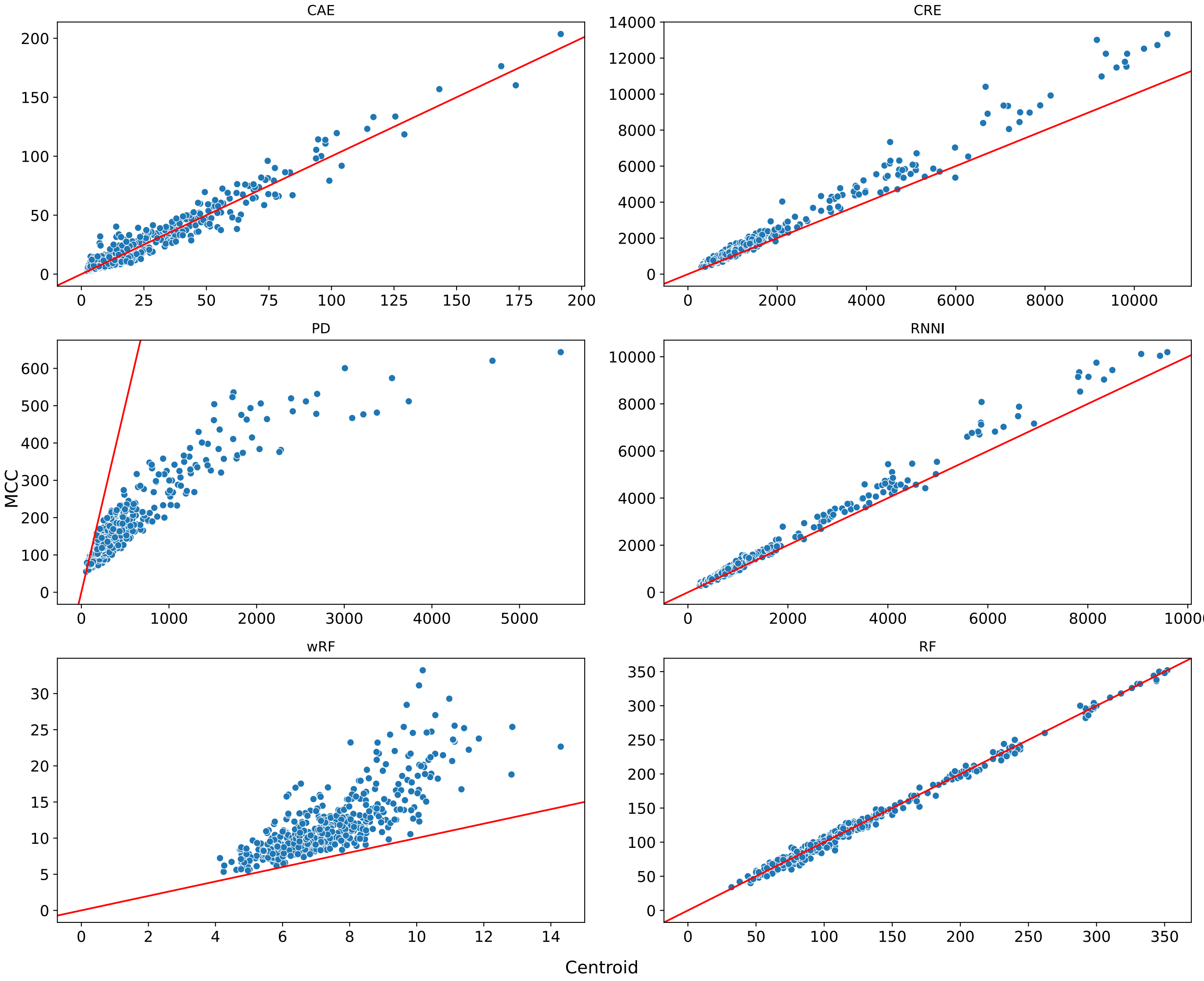

Fig. 18: Comparison of the different error measures in absolute values, the red diagonal is the identity line. The error measures displayed here are clade age error (CAE), clade rank error (CRE), path difference metric (PD) (Steel and Penny, 1993), RNNI metric, weighted Robinson Foulds metric (wRF) and the Robinson Foulds metric (RF). Points above the red line indicate a higher value for the MCC tree and dots below the line indicate higher values for the Centroid tree for the respective measure.

BEAST2 analysis for 1 000 iterations, logging every tree. We run two types of simulations, one of which we refer to as "hill climbing" with a pre-burn-in of 500 samples and the "peak" simulation where we discard 500 000 samples before sampling 1 000 trees. See Figure 7, Figure 24a, and Figure 24c for the "hill climbing" simulations and Figure 23, Figure 24b, and Figure 24d for the "peak" simulations.

For every pair of trees within each simulation we consider the distance between them and plot it against the difference in the two corresponding log likelihoods. Note that both the distance and the difference in log likelihoods are normalised by the largest observed value, respectively.

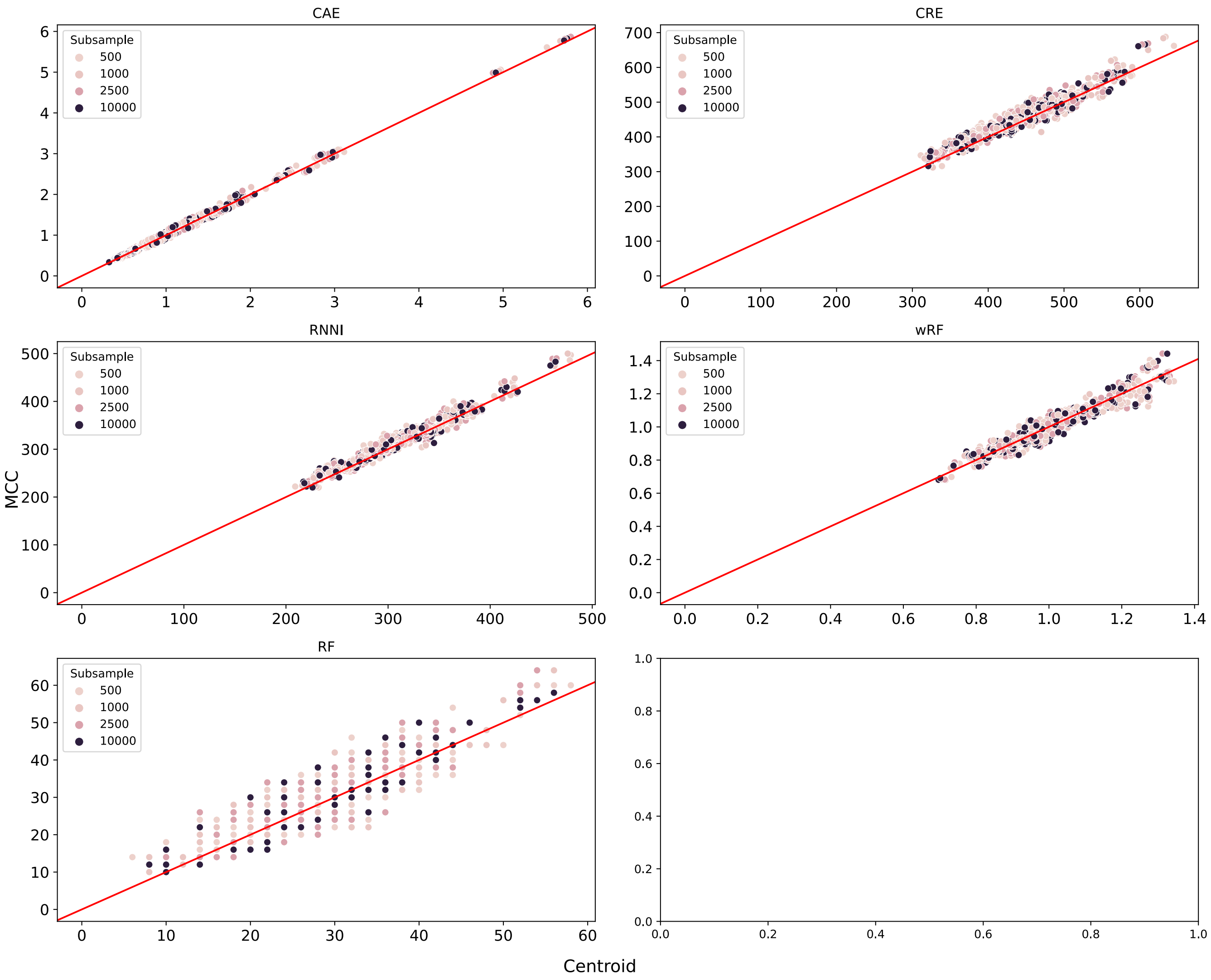

Fig. 19: Comparison of the different error measures in absolute values, the red diagonal is the identity line. The error measures displayed here are clade age error (CAE), clade rank error (CRE), RNNI metric, weighted Robinson Foulds metric (wRF) and the Robinson Foulds metric (RF). Points above the red line indicate a higher value for the MCC tree and dots below the line indicate higher values for the Centroid tree for the respective measure. The different colours represent different sized samples of trees that are taken from the start of 50 000 trees after discarding 1 000 trees as burn-in

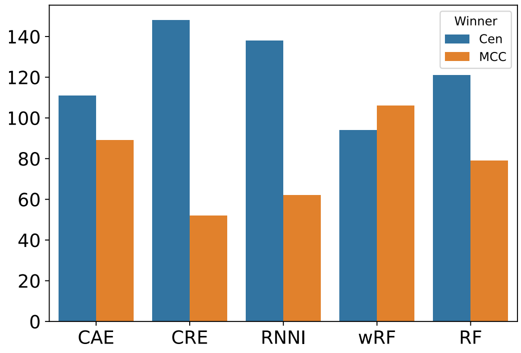

(a) 500 trees

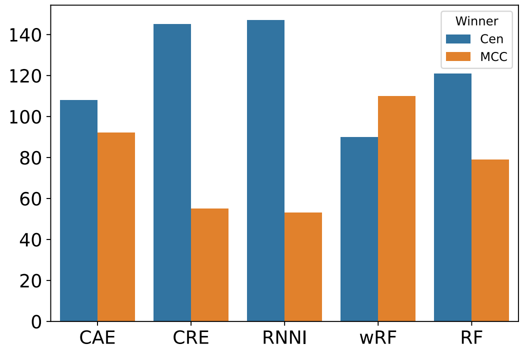

(b) 1000 trees

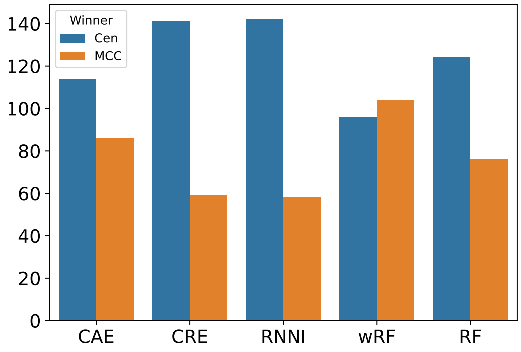

(c) 2500 trees

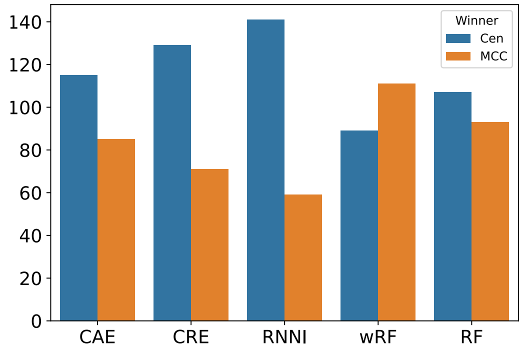

(d) 10000 trees

Fig. 20: Comparison of the centroid approximation and MCC tree using different evaluation measures. The y-axis shows in how many simulations either MCC or the centroid approximation was superior. The error measures are clade ages error (CAE), clades rank error (CRE), RNNI metric, weighted Robinson-Foulds (wRF) and regular Robinson-Foulds metric (RF). All measures compare the summary trees with the true tree for each HKY simulation on different sized subsets after discarding the first 1000 trees as burn-in.

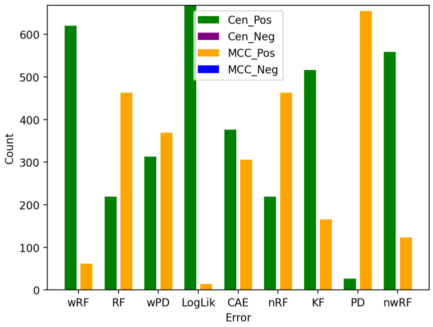

(a) Including datasets with fewer than 50 taxa

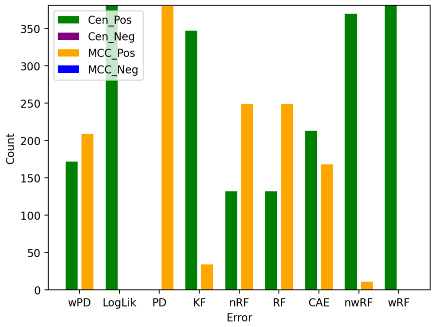

(b) Only datasets with more than 50 taxa

Fig. 21: The error measure comparison slightly changes when small taxa datasets (fewer than 50) are included. This is because for these datasets it can often be the case, especially for data with fewer than 20 taxa, that the MCC tree and our centroid approximation are identical. The plot (A) includes simulations with $10, 12, 15, 20, 25, 30$ and 35 taxa.

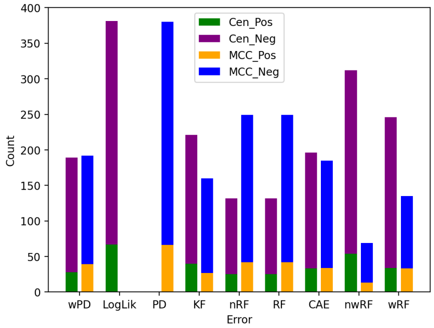

Fig. 22: Comparing the error measures with the median branch length annotation for the MCC tree. Bars are split when the considered MCC tree has negative branch lengths, hence the identifier *Neg* (purple and blue bars) always implies negative branches in the MCC tree for the respective simulated dataset.

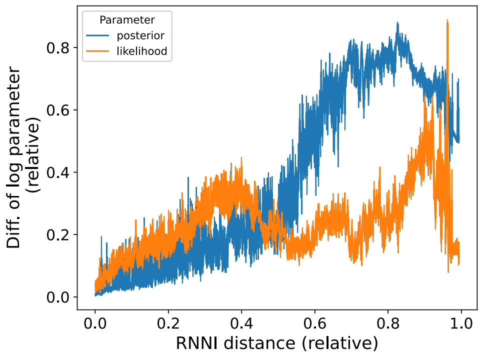

(a) RNNI space

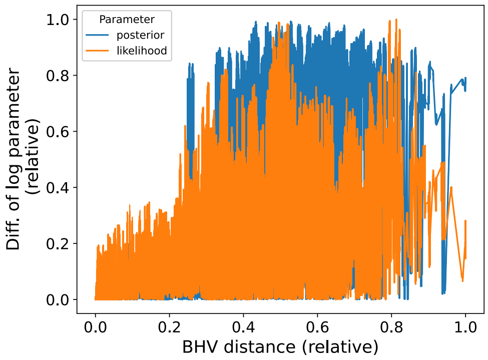

(b) BHV space

Fig. 23: Smoothness assessment for samples from the peak of the distribution, i.e. samples from the end of a MCMC analysis.

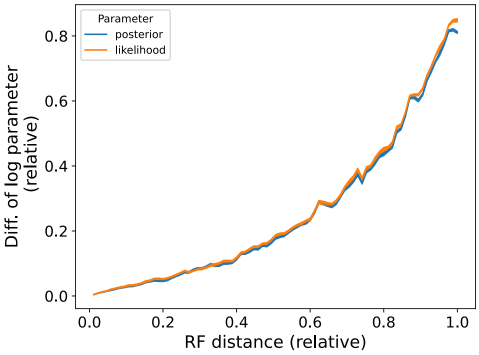

(a) Hill climbing samples for Robinson-Foulds

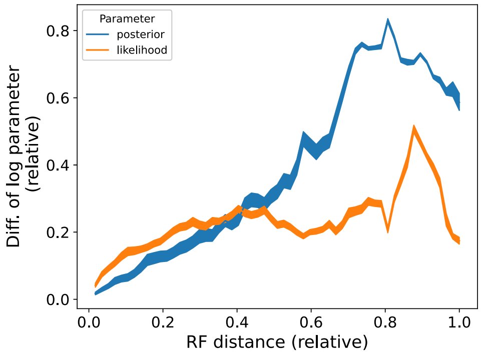

(b) Peak samples for Robinson-Foulds

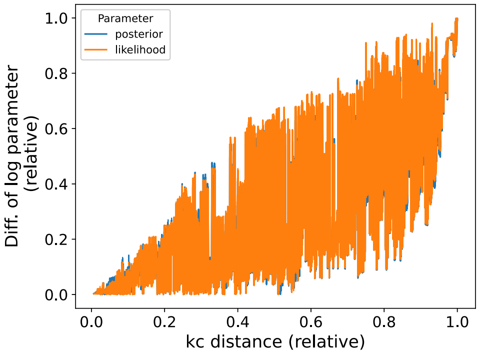

(c) Hill climbing samples for Kendall-Colijn

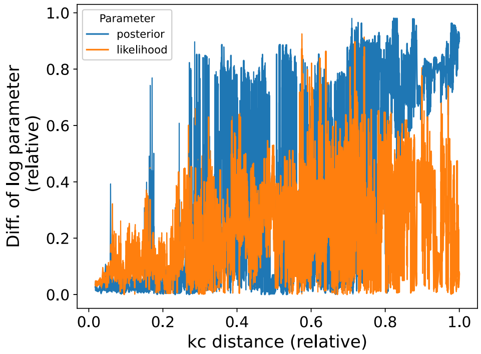

(d) Peak samples for Kendall-Colijn

Fig. 24: Smoothness assessment using the Robinson-Foulds and Kendall-Colijn distance.

Real data application

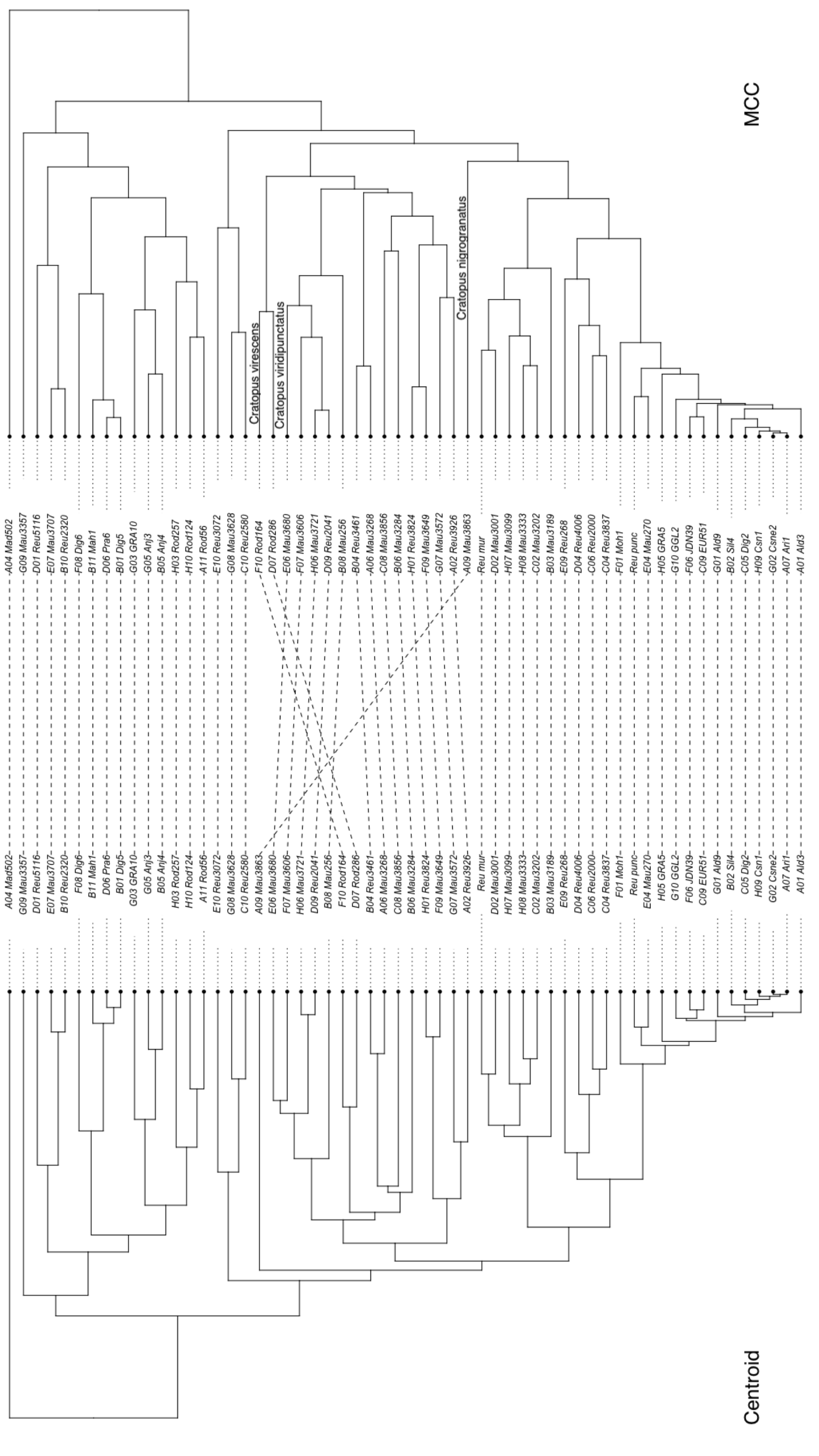

Fig. 25: On this weevil dataset the trees differ in the position of three species as discussed in the main paper.
